# Eco-evolutionary strategies drive viral diversification in nutrient-poor soils across elevation gradients

**DOI:** 10.1093/nsr/nwaf374

**Published:** 2025-09-08

**Authors:** Da Lin, Jianjun Wang, Yu-Qiu Ye, Tian-Lun Zhang, Ming-Ming Sun, Wei-Dong Kong, Long-Jun Ding, Michael R Gillings, Thulani P Makhalanyane, Mao Ye, Dong Zhu, Yong-Guan Zhu

**Affiliations:** State Key Laboratory of Regional and Urban Ecology, Institute of Urban Environment, Chinese Academy of Sciences, Xiamen 361021, China; University of Chinese Academy of Sciences, Beijing 100049, China; State Key Laboratory of Lake Science and Environment, Nanjing Institute of Geography and Limnology, Chinese Academy of Sciences, Nanjing 211135, China; Zhejiang Key Laboratory of Pollution Control for Port-Petrochemical Industry, Ningbo 315830, China; State Key Laboratory of Regional and Urban Ecology, Institute of Urban Environment, Chinese Academy of Sciences, Xiamen 361021, China; University of Chinese Academy of Sciences, Beijing 100049, China; Soil Ecology Lab, Nanjing Agricultural University, Nanjing 210095, China; College of Life Science, Capital Normal University, Beijing 100048, China; University of Chinese Academy of Sciences, Beijing 100049, China; State Key Laboratory of Regional and Urban Ecology, Research Center for Eco-Environmental Sciences, Chinese Academy of Sciences, Beijing 100085, China; School of Natural Sciences, Macquarie University, Sydney 2109, Australia; ARC Centre of Excellence in Synthetic Biology, Macquarie University, Sydney 2109, Australia; Department of Microbiology, Stellenbosch University, Stellenbosch 7602, South Africa; National Engineering Research Center for Soil Nutrient Management and Pollution Remediation, Institute of Soil Science, Chinese Academy of Sciences, Nanjing 210008, China; State Key Laboratory of Regional and Urban Ecology, Institute of Urban Environment, Chinese Academy of Sciences, Xiamen 361021, China; University of Chinese Academy of Sciences, Beijing 100049, China; Zhejiang Key Laboratory of Pollution Control for Port-Petrochemical Industry, Ningbo 315830, China; State Key Laboratory of Regional and Urban Ecology, Institute of Urban Environment, Chinese Academy of Sciences, Xiamen 361021, China; University of Chinese Academy of Sciences, Beijing 100049, China; Zhejiang Key Laboratory of Pollution Control for Port-Petrochemical Industry, Ningbo 315830, China; State Key Laboratory of Regional and Urban Ecology, Research Center for Eco-Environmental Sciences, Chinese Academy of Sciences, Beijing 100085, China

**Keywords:** African mountain, soil viruses, eco-evolutionary strategy, nutrient constraints, climate pressures

## Abstract

As global change intensifies, understanding the eco-evolutionary trade-offs among soil viral communities and the maintenance of their functional traits across environmental gradients is crucial for predicting soil health and ecological functions. Yet how viral communities respond to environmental change remain poorly understood. Using metavirome sequencing along an elevation gradient, which serves as an ideal proxy for environmental variations, we reveal the extensive diversity of viruses and expand the information on soil viruses in Africa. Compared to climate pressures associated with increasing elevation, nutritional constraints driven by higher elevation were more closely associated with significant differentiation in viral populations, mainly driven by an increase in both lytic viruses and functional diversity. These findings were consistently supported by field microcosm experiments on the same mountainsides and the global data sets from other mountain regions. With increasing elevation, phages undergo greater diversifying selection, encoded more bacterial life history strategy genes associated with stress tolerance and ruderals/opportunist, and had a higher proportion of unannotated functions, potentially playing a role in host carbon assimilation in nutrient-poor environments. These findings provide insights into the biogeography and ecological roles of viruses and serve as a foundation for understanding the response of soil viruses to global change.

## Teaser

This work reveals viral eco-evolutionary strategies across elevation gradients, offering new insights into the evolutionary trajectories of soil viruses in response to global change.

## INTRODUCTION

Viruses are the most abundant and widely distributed biological entities in soils and mediate many critical ecosystem services [1,2]. Unraveling interactions between viruses and bacteria is central to predicting the dynamics of microbial communities, and is gaining increased attention [3,4]. Phages (viruses infecting bacteria) can be lysogenic or lytic, based on their infective properties [5]. Lysogenic phages integrate their DNA into the chromosome of their host, replicating and coexisting with the host [6]. These phages can benefit the host by incorporating auxiliary metabolic genes (AMGs) into the host genome, which then contribute to metabolism processes [7–9]. For example, phages in deep-sea, polar, and desert ecosystems have been found to carry AMGs related to carbon metabolism, such as part of the particulate methane monooxygenase gene cluster (*pmoC*) and glutamine synthetase (*glnA*), which help regulate carbon loss under resource-limited conditions [10,11]. In contrast, lytic phages can facilitate the direct return of organic carbon to the food chain by lysing host cells, thereby shortening the organic carbon cycling pathway and creating a unique ‘viral shunt’ in the micro-food web [12]. This process helps maintain microbial biomass in resource-limited environments [13]. Consequently, viruses can help regulate the structure and metabolism of microorganisms, and therefore influence ecosystem processes and biogeochemical cycles [14].

Soils undergo pronounced and frequent challenges, including changes in temperature, rainfall, and nutrient availability. Such challenges are likely to be exacerbated under global climate change [15]. Soil viruses could serve as critical biological regulators that influence microbial survival in response to environmental fluctuations [16,17]. However, the specific impact of adverse environmental conditions, such as climate pressures and nutrient constraints on the ecological and evolutionary dynamics of virus-host interactions at the community level is largely understudied. The viral communities in soils harbor an extensive, uncharacterized diversity of functions, which poses a significant challenge in comprehending the responses of viruses to environmental change [18–20]. The life history strategy scheme offers a practical framework that collapses the complexity of functional diversity into three dimensions that attempt to capture the primary connections and trade-offs among traits. These dimensions are defined as maximizing resource capture (competitor, C), persisting under low resources and stressful conditions (stress tolerance, S), and responding quickly to disturbances (ruderals, R) or facilitate the monopolization of resources (opportunist, O), collectively known as the CSR or CSO strategy [21–23]. These trait-based approaches that incorporate both phenotypic and genotypic information have recently been used to understand the life history strategies of bacteria [24–28]. However, the bacterial life history trait genes encoded by viral communities remain largely unclear, especially in natural habitats under global change.

The goal of this study is to advance our understanding of how soil viral communities respond to environmental gradients, such as climate factors and nutrient conditions, and to uncover the ecological mechanisms driving these responses. Climate factors can directly impact virus activities in soils independently of host feedback [1,15], and long-term warming does not alter the lysogenic-to-lytic ratio of viruses as shown in previous studies [29]. Switching between lysogenic and lytic cycles can be influenced by nutrients, showing that viral communities interact with their hosts in response to resource availability [6,30]. Based on these findings, we hypothesized that (1) with the change of environmental gradient, variations in soil nutrients have a greater impact on the interaction between viruses and hosts compared to climate factors. Additionally, given the potential connection between viral lifestyle and functions [31], we further hypothesize that (2) changes in soil nutrients can alter viral lifestyles and influence viral functions.

**Figure 1.**
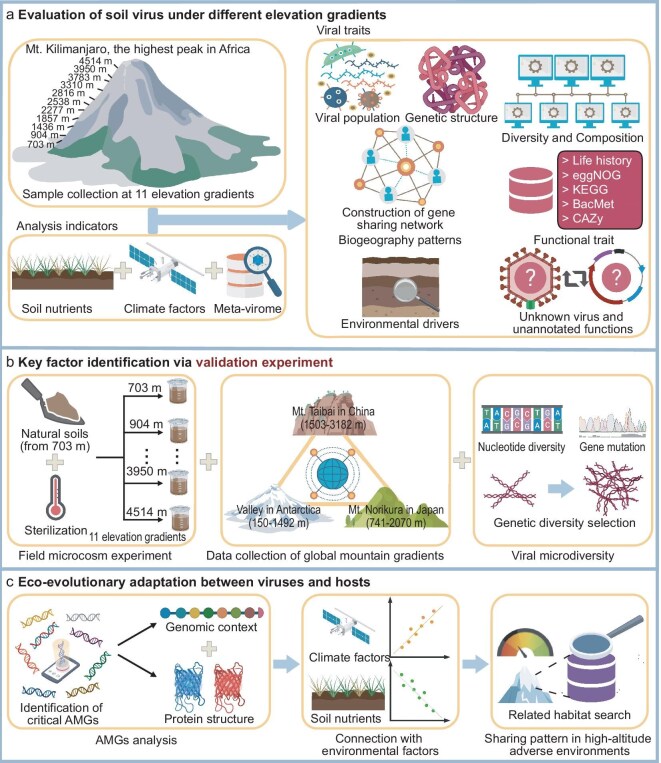
Flow charts depicting the exploration of viral traits, key influencing factors, and eco-evolutionary adaptations between viruses and hosts across different elevation gradients. (a) Our study collected soil samples from Mt. Kilimanjaro, the highest peak in Africa, spanning 11 elevations (703–4514 m). We conducted soil nutrient analysis, gathered climate data, and performed metavirome sequencing to explore the geographic distribution pattern, diversity, composition, functional traits, unknowns, and environmental drivers of viruses. (b) By sterilizing natural soils for field microcosm experiments, gathering data from various global elevation gradients, and examining the microdiversity of viruses, we investigated the key factors that influence viral traits across different elevation gradients. (c) We identified critical AMGs, explored their genomic context and protein structure, elucidated their relationship with climate factors and soil nutrients, and examined the sharing patterns of these AMGs in other high-elevation adverse areas, thereby analyzing the ecological adaptation process of soil viruses and how hosts respond to elevation changes.

To test these hypotheses, we chose Mt. Kilimanjaro as a model location for achieving large-scale environmental gradient changes. Mt. Kilimanjaro, located in East Africa, reaches an elevation of 5892 m, spanning climates from tropical to alpine [32]. Such gradients are commonly used as proxies for studying the impacts of environmental variation on organisms because they encompass a diverse range of climatic zones [33]. Additionally, given the fact that research on soil viruses in Africa is relatively limited, exploring natural habitats could help fill this knowledge gap. Thus, we examined viral communities at 11 elevations (703–4515 m) by metavirome sequencing, established field microcosm experiments at the corresponding elevations, and collected multiple global elevation datasets to support the observed findings. The main objectives were to: (1) investigate changes in soil viral communities and their functional traits with elevation; (2) elucidate key factors driving these changes; and (3) reveal the universal eco-evolutionary adaptation between viruses and hosts under environmental pressure (Fig. 1).

## RESULTS

### Features of soil viral communities

We evaluated two levels of information for viral communities: (1) viral populations represented by viral operational taxonomic units (vOTUs); and (2) the genetic structure of viruses characterized by protein clusters (PCs).

**Figure 2.**
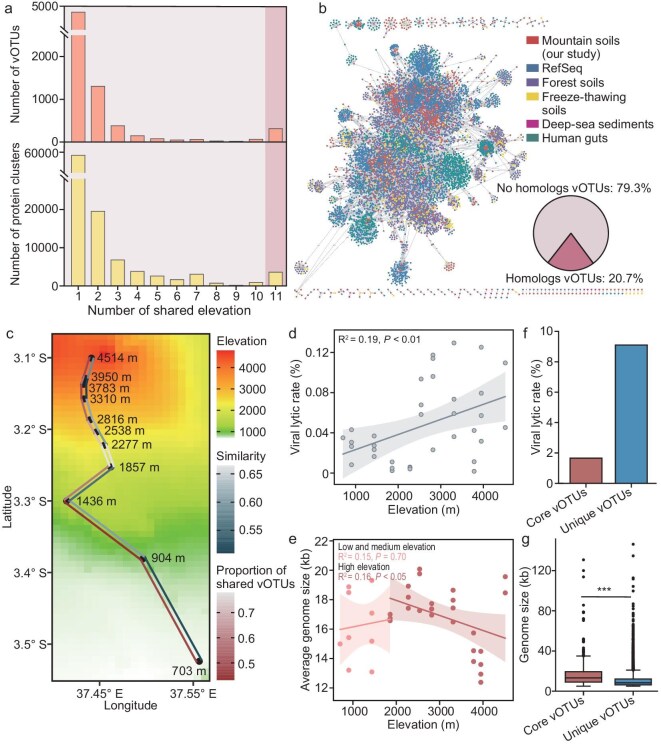
Viral community features across elevation gradients in African mountains. (a) Number of vOTUs (above) and PCs (below) shared among multiple elevations. Core vOTUs referred to those vOTUs shared across all elevations. In contrast, unique vOTUs were derived by merging vOTUs from various samples. (b) Gene-sharing network of viral sequences from our study soils, forest soils, freeze-thawing soils, deep-sea sediment, human gut, and the RefSeq database. Viruses (nodes) are connected by edges, which represent significant pairwise similarity in their shared protein content. The red and pink sections of the pie chart represent the proportion of vOTUs annotated with homologous sequences and those unannotated with homologous sequences in this gene-sharing network, respectively. (c) Calculated connectivity of viral populations between neighboring elevation sites based on the similarity of vOTU profiles and the proportion of shared vOTUs. The color of the solid line reflects the level of connectivity between adjacent elevation sites. Linear regression showed that the relationship between (d) viral lytic rate, (e) the average genome size of the viral communities and elevation in African mountains. Comparison of the (f) viral lytic rate and (g) genome size between core and unique vOTUs.

A total of 6786 vOTUs and 98 096 PCs were identified across 11 elevations (Fig. 2a). Beyond the 10th elevation (3950 m), additional vOTUs and PCs continued to be identified, but also showed a trend towards reaching a plateau, as indicated by accumulation curves (Fig. S1). This suggests that the population and genetic diversity of viruses were relatively well sampled. The majority of vOTUs (*n* = 4260, 62.8%) and PCs (*n* = 55 118, 55.7%) were unique to individual samples as indicated by comparative analysis (Fig. 2a), highlighting the substantial variation in viral populations and their genetic structures across different elevations.

To further investigate the diversity of viral communities, we examined their relationships with viral sequences identified from the reference databases, specific soil habitats (forest soils [34] and freeze-thawing soils [35], considering that our soil samples mainly consist of forests and freeze-thaw soils at high elevations), and other habitats (deep-sea sediments [36] and the human gut [37]). A gene-sharing network was constructed to allocate viral sequences to viral clusters (VCs). Only 1402 vOTUs were clustered into VCs, with the majority (79.3%) lacking homologs in these databases (Fig. 2b).

Across the elevational gradient, viral hosts were mainly composed of Actinobacteria (42.8%) and Proteobacteria (41.7%) (Fig. S2a). A significant portion of vOTUs were classified as members of the Caudoviricetes class, constituting 86.1% of the total identified vOTUs (Fig. S2b). However, 49.0% of vOTUs could not be taxonomically assigned at the family or even higher levels. The proportions and abundances of such unassigned vOTUs increased with elevation (Fig. S2c). These results indicate that most viruses are unique to the environment of Mt. Kilimanjaro and are yet to be described.

To further understand the relationship between elevation and virus diversity, we calculated the connectivity and proportion of shared vOTUs between neighboring elevation samples. These indices showed substantial differences between adjacent elevations at medium to low elevations (below 1857 m), likely due to considerable variation in geographical distance (Fig. 2c). In high-elevation regions (above 1857 m), while the distance between adjacent samples narrowed with increasing elevation, the differences in neighboring viral communities tended to increase (Fig. 2c).

In high-elevation areas, each increase in elevation resulted in a reduction in non-metal nutrient elements (e.g. total carbon (TC), total organic carbon (TOC), total nitrogen (TN), NH_4_^+^-N, total phosphorus (TP), and PO_4_^3–^P), rainfall, and temperature (*P* < 0.05; Fig. S3). Conversely, there is an increase in metal elements (e.g. Al, Ba, Ca, Fe, K, Mg, Mn, and Na) (*P* < 0.05; Fig. S3; Table S1). This shows that viral communities may be exposed to nutrient constraints and climate pressures as elevation increases. However, the number of PCs and vOTUs, proportion of viral lysis, and viral Shannon diversity significantly increased with elevation, while the average genome size of viral communities decreased (*P* < 0.05; Fig. 2d and e; Fig. S4a and b).

Differences between core and unique vOTUs were then investigated. Core vOTUs are those shared across all elevations, while unique vOTUs were derived by merging vOTUs from various samples, representing newly generated viruses as elevation increased. Compared to core vOTUs, these unique vOTUs feature smaller genome sizes, higher lysis rates, and a larger proportion of unknown viruses (*P* < 0.001; Fig. 2f, g; Fig. S4c). These results indicated that increasing elevation could correlate with a greater number of unknown viruses which have small genomes and a higher proportion of lytic lifestyles.

The role of environmental factors in shaping viral communities was then analyzed. Our results revealed that Na, K, and elevation were the most crucial factors affecting the structure of viral communities (*P* < 0.05; Fig. S5). Mean annual precipitation (MAP) was the primary factor influencing changes in soil metal elements and non-metal nutrient elements at varying elevations, which was markedly positively correlated with most metal cations (Ba, Ca, Fe, K, Mg, and Na) (*P* < 0.05) and significantly negatively correlated with most non-metal nutrient elements (TN, TC, TOC, and NH_4_^+^-N) (*P* < 0.01; Fig. S5). Increase in viral lytic rate was primarily influenced by the decrease in P and the increase in K (*P* < 0.05; Fig. S5).

### Functions of soil viruses at different elevations

**Figure 3.**
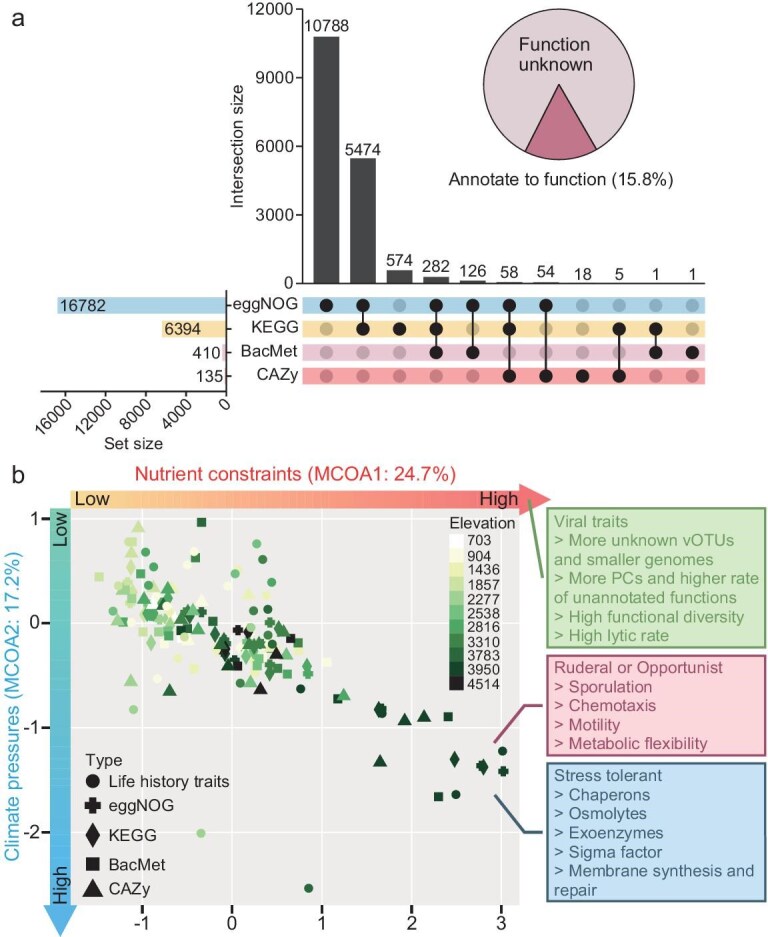
Viral functions across elevation gradients in African mountains. (a) Upset diagrams illustrate the shared functional annotations of viral proteins across the eggNOG, KEGG, BacMet, and CAZy databases. The red and pink sections of the pie chart represent the proportion of viral proteins that are annotated with functional information and those that are not, respectively. (b) Two-dimensional trait space from MCOA analysis showing the functional features of soil viral communities. The red and blue arrows represent the positive direction of the MCOA1 axis and the negative direction of the MCOA2 axis, respectively. Dots represent the positions of the annotated functions of the viral communities used in this study along these two dimensions. Only the bacterial life history trait genes encoded by viral communities, which showed a significant correlation (*P* < 0.05) with the positive direction of MCOA1 and the negative direction of MCOA2, are displayed in the ‘Ruderal or Opportunist’ and ‘Stress tolerant’ trait boxes. Meanwhile, only viral traits with a significant correlation (*P* < 0.05) to the positive direction of MCOA1 are shown in the ‘Viral traits’ boxes.

To explore functional changes in vOTUs with elevation, predicted viral proteins were compared against the eggNOG, KEGG, BacMet, and CAZy databases. The number of genes successfully annotated by the eggNOG, KEGG, and CAZy databases showed a significant positive correlation with elevation, especially in high-elevation areas (*P* < 0.05; Fig. S6). Most viral proteins (84.2%) could not be annotated with corresponding functions (Fig. 3a) and the number of genes successfully annotated from core vOTUs exceeded those from unique vOTUs. Only 13.8% of viral proteins in the unique vOTU set could be successfully annotated with functions, compared to 50.0% in the core vOTU. This indicates that there was an increasing proportion of unannotated gene functions in the viruses from higher elevations (*P* < 0.01; Fig. S7a), suggesting that higher elevation areas may harbor additional, as yet unidentified, functional diversity. The proportion of unannotated genes was positively correlated with the proportion of unclassified viruses (*P* < 0.05; Fig. S7b). AlphaFold3 [38] was used to predict the protein structures of unannotated genes from a representative unclassified vOTU. The majority of protein structures showed high reliability (Fig. S8), implying a strong potential for employing advanced protein interpretation methods to decode unannotated viral proteins in the future.

To better understand how elevation-induced multifactorial changes affect soil viral functions, multitable co-inertia analysis (MCOA) was employed to integrate information from the four (eggNOG, KEGG, BacMet, and CAZy) databases for the above annotation functions, as well as an additional database: life history traits (Fig. 3b). The life history traits database was constructed based on viral genomic functions linked to bacterial life history strategies [27]. In the MCOA analysis, MCOA1 and MCOA2 captured 24.7% and 17.2% of viral function variations, respectively (Fig. 3b). Through bivariate correlation analysis between environmental factors, viral traits (e.g. viral lifestyle, the number of PCs, and the number, Shannon, and abundance of vOTUs), and these two axes, MCOA1 was found to exhibit more significant correlations with soil properties and viral traits (*P* < 0.05; Fig. S9a). Along the positive direction of MCOA1, there was a decrease in soil non-metal nutrient elements (e.g. TC, TOC, and NH_4_^+^-N) and an increase in metal cations (e.g. Na, Ca, Ba, and K) (*P* < 0.05; Fig. S9a). Therefore, the development of this dimension was defined as ‘nutrient constraints’, associated with an increase in the number of vOTUs and PCs, viral functional diversity, and lytic rate (*P* < 0.05). This development also led to the encoding of more bacterial S strategy genes in viral communities, with an increased number of genes encoding chaperons, osmolytes, exoenzymes, sigma factor, and membrane synthesis and repair (*P* < 0.05). Additionally, a trend towards the encoding of more bacterial R or O strategy genes in viral communities was observed, with an increased number of genes encoding sporulation, chemotaxis, motility, and metabolic flexibility (e.g. central/primary metabolism and the degradation of complex polysaccharides such as chitin and glucan) (*P* < 0.05; Fig. 3b; Fig. S9a). MCOA2 showed a stronger link with climate factors. Towards the negative direction of MCOA2, there was an increase in elevation, solar radiation, and latitude, and a decrease in mean annual temperature (MAT) and longitude (*P* < 0.05; Fig. 3b; Fig. S9b). Therefore, this dimensional trend was characterized as ‘climate pressures’, but did not result in changes to viral lifestyles (Fig. S9b).

### Identifying key factors shaping viral diversity along elevation gradients

**Figure 4.**
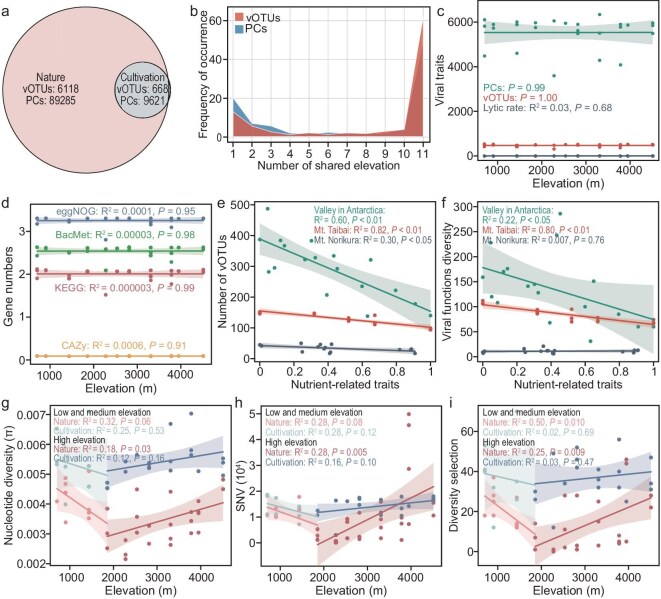
Identification of key factors affecting soil viruses under elevation changes through validation experiments. (a) Venn diagram illustrating the shared patterns of vOTUs and PCs between natural soil and field microcosm cultivated soil. (b) The shared distribution frequency of vOTUs and PCs in cultivated soil at different elevations. (c) Linear regression illustrates the relationship between viral traits (i.e. viral lytic rate, the number of PCs and vOTUs) and elevation. (d) The relationship between the number of annotated functional genes in four databases and elevation in cultivated soils. The relationship between (e) the number of vOTUs, (f) viral functional diversity, and nutrient-related traits in Mt. Taibai, Mt. Norikura, and Valley of Antarctica. The relationship between (g) nucleotide diversity, (h) SNV, (i) diversity selection and elevation in natural soil and cultivated soil.

To further verify the shaping effects of nutrient constraints and climate pressures on viral communities and functions, field microcosm experiments were conducted. Sterilized natural soils from Mt. Kilimanjaro, collected at an elevation of 703 m, were placed at 11 different elevations corresponding to natural soil collection points for cultivation. This ensured consistent nutrient conditions while varying climate factors across the entire experiment. Indeed, after cultivation, there was no significant change in the nutrient content (*P* > 0.05; Tables S2 and S3). Throughout the experiment, due to the fully open cultivation, microbial communities could be transferred from surrounding areas to the cultivated soils through various means, such as fugitive dust or rainfall. Results showed that 96.5% of vOTUs and 99.41% of PCs in the cultivated soils matched 9.8% of vOTUs and 12.09% of PCs found in the surrounding natural soils (Fig. 4a). Additionally, 62.3% of vOTUs and 49.9% of PCs were shared across all elevations (Fig. 4b), and the number of vOTUs and PCs, viral lytic rate, and functional diversity did not significantly change with elevations, MAT, MAP, and solar radiation (*P* > 0.05; Fig. 4c, d; Table S4).

To understand more general patterns in soil virus populations across elevation gradients worldwide, we recovered viral genomes from metagenomic data collected from mountains in various regions. These regions included Mt. Taibai in Shaanxi Province, China [39], Mt. Norikura in Nagano Prefecture, Japan [40], and the Valley in the Shackleton Glacier region, Antarctica [41]. Previous data revealed that soil nutrients in Mt. Taibai and Mt. Norikura, and soil salinization in Antarctica varied with elevation. We performed linear scale normalization on these indicators, adjusting all raw values to a consistent range of 0 to 1, which we refer to as nutrient availability [42], i.e. higher soil nutrient levels in Mt. Taibai and Mt. Norikura, and lower salinization levels in the Valley of Antarctica indicate greater nutrient availability. These normalized nutrient availability values were significantly negatively correlated with the number of vOTUs, functional diversity in the Mt. Taibai and Antarctica, and viral lytic abundance in Antarctica (*P* < 0.05; Fig. 4e, f; Fig. S10).

Nucleotide diversity (π), single nucleotide variants (SNVs), and pN/pS ratios (ratio of non-synonymous to synonymous polymorphisms) were calculated to track viral microdiversity [43]. The nucleotide diversity and SNVs of viruses showed an increasing trend with elevation in natural soils (*P* < 0.05) but not in cultivated soils (*P* > 0.05) (Fig. 4g, h). These results illustrated that nutrient constraints can also contribute to an increase in viral microdiversity and gene mutations. Further exploration revealed that viral genes putatively undergoing diversifying selection (pN/pS >2.5) [3] increased with elevation only in natural soils (*P* < 0.01), whereas no such trend was observed in cultivated soils (*P* > 0.05; Fig. 4i). These viral genes experiencing diversifying selection (55.1% of a total of 554) in natural soils could be annotated with functions, with 28.0%, 20.5%, and 6.5% associated with metabolism; DNA replication, recombination, and repair; and transcription, respectively (Fig. S11).

### Eco-evolutionary adaptation between viruses and hosts under environmental pressure

**Figure 5.**
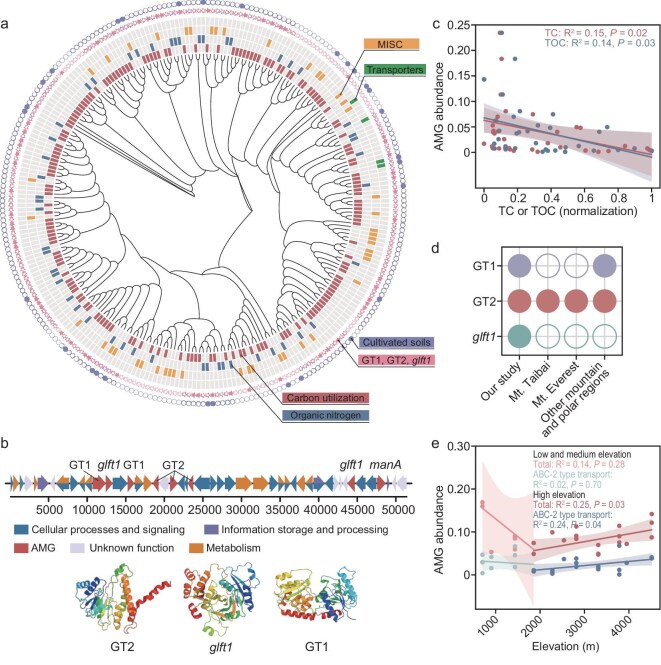
Genomic analysis of ecological adaptation patterns between viruses and hosts. (a) Phylogenetic tree showing the maximum likelihood of viruses carrying putative AMGs. The colors on the outer side represent AMGs related to carbon utilization (red), organic nitrogen (blue), MISC (yellow), and transporters (green), including glycosyltransferase genes: GT1, GT2, and *glft1* (pink), and AMGs identified in cultivated soil (purple). (b) Genomic context and protein structure of viral genomes containing GT1, GT2, and *glft1* genes. (c) Linear regression illustrates the relationship between TC, TOC, and key AMGs related to carbon utilization (GT1, GT2, and *glft1*) in natural soil. (d) The occurrence of GT1, GT2, and *glft1* genes in other high-elevation adverse environments. (e) The relationship between AMG abundance and elevation in cultivated soil.

Investigation into auxiliary metabolic genes (AMGs) encoded by vOTUs was conducted to better understand the impact of viral genes on bacterial host metabolism and biogeochemical cycles. A total of 314 genes were identified as putative AMGs, categorized into four groups based on their functions: carbon utilization, MISC (miscellaneous), organic nitrogen, and transporters (Fig. 5a). The abundance of these AMGs exhibited a considerable positive correlation with increasing elevation, whether considering unique vOTUs, core vOTUs, or overall vOTUs (*P* < 0.05; Fig. S12a, b). Among these, the most significantly increased AMGs were those related to carbon utilization, which were widely distributed in phages and clustered phylogenetically (Fig. 5a).

To further understand its functions and specific impact on host metabolism, representative phage encoding AMGs for carbon utilization was assembled. The most common phage genes encoded glycosyltransferases (GT1, GT2, and *glft1*) (Fig. 5b). These three genes were found in 68 predicted phage genomes, showing a notable positive correlation with elevation in high-elevation regions (Fig. S12c). They constituted almost 40% of the total AMGs, and their overall abundance significantly negatively correlated with TC and TOC concentrations (*P* < 0.05), but not with MAT, MAP and solar radiation (*P* > 0.05; Fig. 5c; Fig. S13). A few studies have clearly demonstrated AMGs under high-elevation adverse conditions. In particular, glycosyltransferase genes, such as GT2, have been detected on Mt. Taibai, Mt. Everest [44], and in other mountainous and polar regions [45] (Fig. 5d).

Furthermore, the abundance of AMGs in cultivated soils, with constant nutrient levels across elevations, still notably increased with elevation in high-elevation regions (*P* < 0.05; Fig. 5e). A total of 36 AMGs were detected in the cultivated soils, all of which were also identified in natural soils (Fig. 5a). Unlike in natural soils, transporters—represented by ABC-2 type transport protein (*pcsA*), which accounted for 28.0% of the total AMG abundance—showed a more pronounced increase with elevation (Fig. 5e). In contrast, the three glycosyltransferases GT1, GT2, and *glft1* significantly increased in nutrient-poor natural soils (*P* < 0.05), but did not change with elevation in nutrient-invariant cultivated soils (*P* > 0.05; Fig. S14).

## DISCUSSION

This study revealed diversity in the population and genetic structure of viruses at varying elevations on Mt. Kilimanjaro. Although the soils on this mountain contained some viruses found in other soil habitats and the human-related viral communities, the majority of viruses in this environment were unique and previously unrecognized. This trend may become more pronounced with increasing elevation. Consequently, significant and novel viral diversity could be associated with the extreme conditions typical of high-elevation regions, such as limited nutritional availability, low temperatures, frequent freeze-thaw cycles, and intense solar radiation [46,47]. These findings, combined with the relative lack of soil viral data for the African region [48,49], highlight the urgent need to explore the uncharted ‘dark matter’ of soil viruses on a regional scale in Africa. Here, we present a viral genomic catalog of soils in an African mountain, which fills some of the existing gaps in our understanding of African soil viruses.

After examining the distribution patterns and driving factors of samples across different elevations, we observed that elevation played a crucial role in shaping the viral communities, particularly in higher elevation areas. These changes in elevation primarily generate shifts in climate factors and soil nutrition. High elevations result in climate pressures characterized by low temperatures and reduced rainfall. Long-term low temperatures and rainfall lead to sparse vegetation and increased aridity [50]. This, in turn, could cause a decrease of soil nutrients and an increase in salinity, ultimately resulting in nutrient constraints [51,52]. Under these adverse circumstances of climate pressures and nutrient constraints, there was an unexpected increase in the viral lytic rate, accompanied by a rise in viruses with smaller genomes and the increased diversity of viral communities. Through MOCA analysis and subsequent field microcosm experiments, we showed that this trend could be primarily attributed to nutrient constraints. Unlike bacteria, which generally exhibit larger genome sizes and lower species diversity under oligotrophic conditions [27,53,54], viruses displayed distinct responses. This phenomenon may be attributed to the unique ecological strategy of viruses, consistent with previous research that lower nutrient levels in deeper soils increased the prevalence of lytic viruses [29]. The rapid lifecycle of lytic viruses [55] may lead to the production of more viruses with smaller genome sizes, most of which cannot be recognized, thereby enriching population diversity. Moreover, reports exist of the ‘viral shuttle’ in nutrient-poor soils [56], which plays a key role in the nutrient cycling of micro-food web. This partly support the notion that nutrient constraints promote the abundance of lytic viruses. Specifically, phages contribute host-derived carbon to the soil organic carbon pool by lysing host cells. As host cell materials are released into the soils, they serve as important substrates for the growth of other microbial populations, thereby accelerating the organic carbon cycling processes, alleviating nutrient limitations, and supporting the survival of microbial communities [55].

In addition, the proliferation of these viruses with smaller genome sizes may also contributes to an increase in functional diversity, which is marked by a greater prevalence of genes encoding bacterial S and R/O strategies. These phages may hijack additional host metabolic genes to encode functions that promote both viral and host survival [31,57]. These include regulating activities (sigma factors) [58], responding to beneficial conditions (chemotaxis and motility) [59], enhancing tolerance to nutrient-poor environments (sporulation) [21,60], resisting environmental stress (osmolytes, chaperons, membrane synthesis and repair) [61–63], and effectively utilize carbon sources. Overall, the functional traits of viruses under nutrient-poor environments in our study align with the versatility and characteristics observed in bacterial taxa as described in previous articles [23,27]. Additionally, high-elevation nutrient-poor regions contain more unannotated genes, likely stemming from a higher presence of unclassified viral communities [53], and could be expected to further strengthen our conclusion regarding the increase in viral functional diversity. This observation also suggests that high-elevation nutrient-constraint regions may serve as reservoirs for a broad range of uncharacterized functions.

We further conducted *in situ* soil transplantation experiments employing sterilization treatments that controlled for nutrient content in soils, thus focusing solely on the impacts of climate factors. We observed that nearly all viruses and their genetic structure in the cultivated soil matched those in the original natural soils. However, after one month of cultivation, we note significant homogeneity across different elevations, with >60% of viruses being distributed consistently, and viral lytic rate and functional diversity did not change with climate pressures, which may indirectly suggest the influence of nutrient constraints. Furthermore, we collected published metagenomic data from three additional mountains characterized and recovered viral genomes. This revealed a negative correlation between viral diversity, lytic abundance, functional diversity and soil nutrient levels along these elevation gradients, in accordance with our results. These data potentially supported our hypothesis, that compared to climate factors, variations in soil nutrients may have a greater impact on viral lifestyles, thereby affecting the traits of viral functions. Upon extensive analysis, we discovered that heightened nutrient constraints alter the microdiversity of viruses. This was supported by increased nucleotide diversity, gene mutations, and genes undergoing selection for diversity, primarily related to maintaining DNA integrity and expediting the biosynthesis of viral components [64]. These findings suggest a potential genomic evolutionary mechanism by which nutrient constraints enhance functional diversity [65].

So far, there is very limited information available on soil viral AMGs in high-elevation environments [45,64]. Our results identified numerous predicted AMGs that exhibit enrichment with increasing elevation. After examining the similarities and differences of AMGs in natural and cultivated soils, and their relationship with environmental factors, we found that both nutrient constraints and climate pressures likely promote accumulation of phage AMGs. In nutrient-poor environments, AMGs related to carbon utilization, represented by the glycosyltransferase genes GT1, GT2, and *glft1*, are more prevalent and significantly increased. Meanwhile, the identification of glycosyltransferase genes, notably the GT2 gene, in multiple high-elevation mountainous habitat [44,45] and their presence in various soil environments [29,66] as previously reported, provides evidence for the potential broad distribution of these phage AMGs across different soil habitats, including extreme environments. Furthermore, climate pressures led to an increase in the abundance of AMGs associated with ATP biosynthesis.

The above findings reveal the response patterns of viruses in soil ecosystems to environmental changes, particularly the complex ecological adaptation strategies under nutrient constraints. These strategies may differ from those observed under chemical stress, though in both cases, viruses adapt to challenging environmental conditions by modifying their interactions with hosts. In our study, under nutrient constraints, viruses may contribute to nutrient cycling by breaking down host cells and enhancing AMG related to nutrient metabolism. In contrast, studies on chemical pollution showed that chemical pollutants can directly impair viruses and negatively impact their diversity, unlike nutrient constraints [67,68]. Viruses, in turn, may help microbial communities adapt to chemical stress by strengthening their symbiotic relationships with hosts and carrying AMGs related to basic metabolism and the biodegradation of chemical pollutants [67,68]. This allows viruses to harness microbial environmental resistance and avoid direct exposure to harsh conditions. In addition, when facing with nutrient constraints, similar adaptive trends of virus, which play important roles in element cycling by compensating or enhancing host metabolism, may be observed in both soils and aquatic ecosystems. However, compared to aquatic ecosystems with relatively simple nutritional components, viruses in soil environments with complex polysaccharides—particularly those derived from plants and animals—may accumulate more AMGs related to the degradation of complex carbon sources [14], such as the GT1, GT2, and *glft1* genes mentioned above.

**Figure 6.**
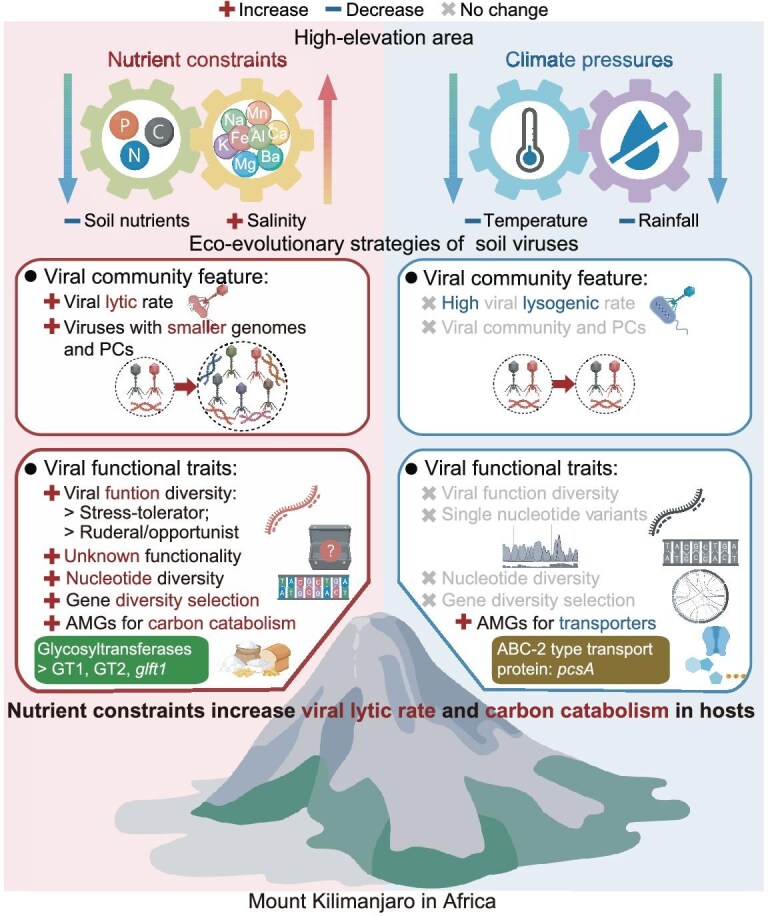
Conceptual model. Conceptual model illustrating the mechanisms by which elevation change induces shifts in soil viral eco-evolutionary strategies.

Our study provides the first evidence on the effects of elevation on the assembly of local viral communities, revealing distinct evolutionary trajectories of soil viruses under nutrient constraints and climate pressures as elevation changes. Nutrient constraints may lead to more complex interactions between viruses and hosts, represented by the emergence of more viruses with smaller genomes and higher lytic rate, which increases the population and functional diversity of the viral communities. Meanwhile, the viral communities hold potential for enhanced host carbon utilization (Fig. 6). Moreover, climate pressures could cause viruses to integrate their DNA into hosts, which may shape the symbiotic physiological state of host to enhance resistance to stress (Fig. 6). This study enhances our understanding of the viral eco-evolution in response to environmental pressures and highlights their role in regulating soil microbial communities and their functions in the environment.

Although this research provides an in-depth analysis of the ecological adaptation strategies of soil viral communities across elevation gradients, it is important to note the potential caveats regarding our research design and metavirome analysis. First, the accurate estimation of viral genome sizes may be affected by the limited capacity of metavirome sequencing, such as the short-read assembly, amplification bias in the viral fraction, and the genetic diversity of viruses. Second, the virus-host dynamics could be partly revealed by our one-month incubation of the cultivated soil experiment, but longer-term cultivation is encouraged for future studies to examine viral communities shifts and further long-term adaptive responses. Third, consistent metavirome sequencing across mountains is encouraged in future studies to offer a more advanced and high-resolution method for characterizing viral communities by involving preprocesses to separate virions from larger microbes. However, due to the lack of metavirome research in other mountains, we opted to recover viral genomes from metagenomics to also offer a consistent characterization of viruses compared to metavirome sequencing [69]. Nonetheless, we should note that some hard-to-recover viruses may be overlooked by the metagenomics, and future studies are encouraged to use consistent metavirome sequencing across mountains to further validate our main findings. Finally, we expect that future multidisciplinary and multiscale studies could be employed to fully understand and assess the ecological and evolutionary dynamics of viruses in the context of global change.

## METHODS

### Study area and field sampling

Our study was performed on the southern and southeastern slopes of Mt. Kilimanjaro in Tanzania, East Africa (3°6'–3°31' S, 37°24'–37°33' E). Mt. Kilimanjaro rises from the savannah plains at 700 m to a snowcapped summit reaching 5895 m [70,71]. Precipitation follows a bimodal pattern, with the MAP peaking around 2200 m. The primary rainy season occurs from March to May, with variable short rains typically in November. MAT decreases steadily with increasing elevation [70,71]. Since 2006, forests located above 1800 m have also been included in the National Park. The soils in these forest zone consist of Andosols with folic, histic, or umbric upper horizons [32,70].

We collected soil samples with replicates from 11 elevation locations ranging from 700 m to 4515 m during February–March 2020, with an average elevation difference of 381 m between each location, i.e. 703, 904, 1436, 1857, 2277, 2538, 2816, 3310, 3783, 3950, and 4514 m. Four replicate samples were collected for each elevation, yielding a total of 44 soil samples. Ultimately, 35 samples were successfully tested for all indicators. To minimize systematic errors and batch effects, and to ensure that this would not affect our research, we did not conduct additional measurements. The samples information available in Table S1. These samples were subsequently divided into two portions: one portion was air-dried for soil physicochemical measurements, and the other was preserved at −80°C for DNA extraction.

### Characterization of soil physicochemical variables and climate factors

Soil physicochemical variables and climate factors were measured and collected to investigate the underlying driving factor for variations viral in communities. After combustion, the TOC, TC, and TN content of soil samples were determined using an elemental analyzer (Elementar Macrocube, Germany) [71]. The total phosphorus (TP) and PO_4_^3−^-P content of the soils were quantified using the alkali fusion-molybdenum antimony anti-spectrophotometric method [72]. The content of soil dissolved inorganic nitrogen (NH_4_^+^-N, NO_X_^−^-N, and NO_2_^−^-N) were measured by a flow injection analyzer (AutoAnalyzer 3 SEAL, Germany)[73]. Metal elements (Al, Ba, Ca, Fe, K, Mg, Mn, and Na) were analyzed by digesting soil samples in a HCl-HNO_3_-HF solution (2:3:1, v/v/v) using a MARS6 microwave system (CEM, USA), followed by measurement using inductively coupled plasma mass spectrometry (ICP-MS) with an Agilent 7500a system (USA) [74].

We retrieved climate factors, i.e. MAT and MAP from the earlier study conducted on Mt. Kilimanjaro [71]. This study included fitting models for MAT (linear fit: *R*^2^ = 0.99), MAP (third-order polynomial fit: *R*^2^ = 0.98), and elevation in 52 locations. The solar radiation data are an acquisition from WorldClim2 (https://www.worldclim.org/).

### Virome sequencing and assembly

To extract soil viral DNA, virus-like particles (VLPs) from samples were initially purified and concentrated using filtration and centrifugation, following a previously established protocol [67,75]. Before viral DNA extraction, virus concentrates were incubated with 10 units of DNase I (Thermo Fisher Scientific, USA) at 37°C for 1 hour to eliminate exogenous DNA. Subsequently, the VLP suspension was utilized for viral DNA extraction using the Takara MiniBEST Viral RNA/DNA Extraction Kit Ver. 5.0. DNA extracted from the viral fraction was amplified using multiple displacement amplification (MDA) to create shotgun metagenomic libraries. Paired-end sequencing (150 bp) was conducted on the HiSeq X platform (Illumina, San Diego, CA, USA).

Following the removal or trimming of low-quality reads using Trimmomatic v0.39, which included removing adapters, bases with quality scores <20, and reads <50 bp, the remaining high-quality reads were then employed for sequence assembly using MEGAHIT v1.2.9 [76]. We evaluated the assembly performance using QUAST v5.2.0 [77] and selected assembled contigs >5000 bp for subsequent bioinformatic analysis.

### Identification, classification, functional annotation, and microdiversity of virus

Viral sequences were identified from the assembled contigs using a combination of DeepVirfinder (v1.1) [78] with a score of ≥0.7 and *P* value ≤ 0.05, Virsorter2 (v2.0) [79] with a minimum score ≥0.50, and VIBRANT (v1.2.1) [80]. All contigs meeting the criteria of 95% average nucleotide identity (ANI) and 85% alignment fraction (AF) were then clustered into viral populations (vOTUs) using CD-HIT v4.6 [81]. The completeness and contamination of these vOTUs were then estimated using CheckV v1.0.1 (database v1.2) [82]. After mapping clean reads from each sample to all vOTUs using Bowtie2 v2.5.0 [83], read counts were normalized to per kilobase per million mapped reads (RPKM), allowing the relative abundance of vOTUs to be expressed across samples. Two methods were utilized for taxonomic classification of vOTUs: vOTUs were taxonomically classified using VPF-Class with a membership ratio (MR) ≥0.5 and a confidence score (CS) ≥0.2 [84]; Predicted Open Reading Frames (ORFs) from the vOTUs were aligned against the NCBI viral_Refseq database using BLAST+ v2.9.0 [85] (e-value ≤10^−^^5^ and bitscore ≥50). The obtained ORFs were translated into proteins and then those with a clustering threshold of >90% were converted to non-redundant viral PCs using CD-HIT v4.6 [81].

The shared network generated by vConTACT3 [86] was visualized using Cytoscape v3.8.0 to illustrate the overlap of viruses found in the African mountain soils with viral genomes sourced from diverse environments, i.e. deep-sea sediments [36], the human gut [37], freeze-thawing soils [35], forest soils [34], and the RefSeq database. When it came to functional annotation, the protein-coding sequences were individually compared against several databases, including the Kyoto Encyclopedia of Genes and Genomes (KEGG), carbohydrate-active enzymes (CAZy), evolutionary genealogy of genes: Non-supervised Orthologous Groups (eggNOG), antibacterial biocide and metal resistance genes database (BacMet), and Comprehensive Antibiotic Resistance Database (CARD) using BLASTP (v2.9.0+) [87], with a threshold of 50 for the bit score and 10^−^^3^ for the E-value. AlphaFold3 [38] was using to model the protein structure of unannotated viral proteins.

Referring to the genomic features related to bacterial life history strategies reported in previous studies [27] and the standards set by previous research [21–23], a life history traits database was established by incorporating the functional classifications of viral genes from the KEGG, CAZy, eggNOG, and CARD databases. For this database, life history trait genes encoded by viral communities were calculated by summing the relative abundances of genes linked to basic metabolism, chaperons, chemotaxis, carbon source degrading enzymes, membrane synthesis and repair, osmoles, sigma factor, sporulation, uptake system, motility, antibiotic resistance, and nutrient metabolism (carbon, nitrogen, and sulfur metabolism) (see details in Tables S5–S8).

Nucleotide diversity (π), single nucleotide variants, and selection measures (the ratio of non-synonymous to synonymous polymorphisms) for each gene in the flagged vOTUs were calculated using inStrain v1.5.3 [88]. Genes were considered under diversifying selection if the pN/pS ratio was >2.5.

### Interaction analysis between viruses and hosts

The lifestyle of vOTUs was predicted using the two following pipelines: (1) Deephage v1.0 was utilized to classify between lytic and lysogenic virus-derived sequences with a cutoff of 0.8 [89]; (2) functional annotation was performed for all ORFs of vOTUs against Pfam v35.0 using ‘hmmscan’ in HMMER v3.1b2 [90] with an e-value threshold of <10^−^^5^. Subsequently, we selected sequences containing lysogenic marker proteins (e.g. transposase, integrase, kinase, lyase, and recombinase proteins) for identification as lysogenic viruses. The vOTUs identified by the pipelines mentioned above were classified as either lytic or lysogenic, while others were considered unknown.

Virus-encoded AMGs were identified using DRAM-v (v1.2.0) [91] in combination with VIBRANT [80], employing default parameters. To mitigate potential false positives stemming from host contamination, only putative AMGs situated between two viral genes or adjacent to viral genes were considered as viral AMGs for subsequent analysis. Phyre2 (v2.0) [92] was utilized to search for secondary and tertiary structural homology and to model the protein structure of AMGs.

Virus-host linkages were predicted using iPHoP [93], which integrates six available virus-host relationship prediction methods (Blast, CRISPR, VirHostMatcher, WIsH, PHP, and RaFAH) and constructs a machine learning framework for comprehensive virus-host prediction. Finally, virus-host linkages with confidence scores higher than 90 were selected for further analysis.

### Validation experiment

#### Field microcosm experiment

The experiment was conducted by using 20 g of soils per sterile beaker. These were sourced from natural soils from Mt. Kilimanjaro at a height of 703 m, to ensure that the nutrient conditions in the microcosms matched those of natural soils. The soils were sterilized three times at 121°C for 4 hours to eliminate potential influences from microbial communities. Meanwhile, sterilized MilliQ water and the following salts were added: CaCl_2_ 7.55 g/l, MgSO_4_·7H_2_O 6.78 g/l, and NHCO_3_ 3.53 g/l to ensure sufficient moisture and nutrients in the cultivated soils. We placed these beakers with their openings exposed at 11 elevations (703, 904, 1436, 1857, 2277, 2538, 2816, 3310, 3783, 3950, and 4514 m), corresponding to natural soil collection points for undisturbed cultivation. Since the same soil source was used but placed at different elevations, the overall experiment maintained consistent nutrient conditions while varying climate factors, including MAT, MAP, and radiation. This setup aimed to explore the impact of climate pressures on soil viral communities and verify the role of nutrient constraints. After 1 month of cultivation, a total of 26 soil samples, with the elevation information for each sample provided in Table S2, were used for nutrient content determination (TOC, TN, and TP), metavirome sequencing, and analysis as described above.

#### Collection of global elevation gradient dataset

We performed a comprehensive literature search using the Web of Science database (http://apps.webofknowledge.com) with the keywords ‘elevation’ and ‘soil’. The following criteria were applied to select relevant studies and samples: (1) only paired-end sequencing reads in FASTQ format were included, and the data size of each metagenomic sample needed to exceed 1 Gb (measured in base pairs); (2) samples were excluded if they were collected near a region that has experienced significant climate change, human pollution, been involved in experimental treatments, or had unclear information; (3) given the research purpose of this article, relevant papers needed to provide detailed results on soil nutrition and include five or more elevation gradients. Finally, we collected a total of 49 metagenomes from Mt. Taibai in Shaanxi Province, China; Mt. Norikura in Nagano Prefecture, Japan; and the Valley in the Shackleton Glacier region, Antarctica, for subsequent identification and analysis of viral genomes (>5000 bp) using the methods described above. Detailed information about the collected metagenomes can be found in Table S9.

### Statistical analyses

The similarity of viral populations between neighboring elevation sampling sites was assessed by calculating the Bray–Curtis distance matrix for each sample, which was then converted to similarity values using the formula: similarity value = 1/(1 + distance matrix) [94]. The proportion of shared vOTUs between neighboring elevation sampling sites was calculated using the following equation [34]:


\begin{eqnarray*}
&&{\mathrm{Proportion\ of\ shared\ vOTUs }}\\ &&\qquad= \ ( ( {{\mathrm{Sn}}/{\mathrm{a}}} ) + \ ( {{\mathrm{Sn}}/{\mathrm{b}}} ) )/2,
\end{eqnarray*}

where ‘a’ represents the number of vOTUs in one and ‘b’ another sample, respectively, and ‘Sn’ represents the number of shared vOTUs between samples ‘a’ and ‘b’.

To integrate functional information of soil viral communities, MCOA was used as an exploratory method that combines data from five functional databases: life history traits, eggNOG, KEGG, BacMet, and CAZy. This approach identifies co-relationships between the databases and applies a covariance optimization criterion to summarize the shared information across multiple multivariate (e.g. omic) datasets [27]. All data were *z*-score transformed to improve normality. The MCOA analysis was performed using the R package ‘ade4’ [95]. The sample coordinates on the first and second dimensions of the MCOA were extracted and used as latent variables representing viral community positions within the functional trait space. Subsequently, bivariate Spearman correlation analysis was conducted to explore the relationships between environmental factors, viral traits, bacterial life history trait genes encoded by viral communities, and these coordinates.

## Supplementary Material

nwaf374_Supplemental_Files

## Data Availability

Metavirome sequencing data produced in this study were deposited in the National Center for Biotechnology Information (NCBI) Sequence Read Archive (SRA) database under accession number PRJNA1163297.
