# Supplementary material for: Eco-evolutionary strategies drive viral diversification in nutrient-poor soils across elevation gradients
Source: Natl Sci Rev. 2025 Sep 8;12(10):nwaf374. doi: 10.1093/nsr/nwaf374 (PMC12501426; doi:10.1093/nsr/nwaf374)
Supplement: nwaf374_Supplemental_Files [file nwaf374_supplemental_files.zip › Supplementary Figure.docx]

**Eco-evolutionary strategies drive viral diversification** **in** **nutrient-poor soils across elevation gradients**

Da Lin^1,2,#^, Jianjun Wang^3,#^, Yu-Qiu Ye^4^, Tian-Lun Zhang^1,2^, Ming-Ming Sun^5^, Wei-Dong Kong^6^, Long-Jun Ding^2,7,*^, Michael R. Gillings^8,9^, Thulani P. Makhalanyane^10^, Mao Ye^11^, Dong Zhu^1,2,4,*^,Yong-Guan Zhu^1,2,4,7^

^1^ State Key Laboratory of Regional and Urban Ecology, Ningbo Observation and Research Station, Institute of Urban Environment, Chinese Academy of Sciences, Xiamen 361021, China;

^2^ University of Chinese Academy of Sciences, 19A Yuquan Road, Beijing, 100049, China;

^3^ State Key Laboratory of Lake Science and Environment, Nanjing Institute of Geography and Limnology, Chinese Academy of Sciences, Nanjing, China;

^4^ Zhejiang Key Laboratory of Pollution Control for Port-Petrochemical Industry, CAS Haixi Industrial Technology Innovation Center in Beilun, Ningbo 315830, China;

^5^ Soil Ecology Lab, Nanjing Agricultural University, Nanjing 210095, China;

^6^ College of Life Science, Capital Normal University, Beijing 100048, China;

^7^ State Key Laboratory of Regional and Urban Ecology, Research Center for Eco-Environmental Sciences, Chinese Academy of Sciences, Beijing, 100085, China;

^8^ School of Natural Sciences, Macquarie University, Sydney, New South Wales 2109, Australia;

^9^ ARC Centre of Excellence in Synthetic Biology, Macquarie University, Sydney, New South Wales 2109, Australia;

^10^ Department of Microbiology, Faculty of Science, Stellenbosch University, Stellenbosch, South Africa;

^11^ National Engineering Research Center for Soil Nutrient Management and Pollution Remediation, Institute of Soil Science, Chinese Academy of Sciences, Nanjing 210008, China;

# Da Lin and Jianjun Wang contributed equally to this work.

*Correspondence should be addressed to: dzhu@iue.ac.cn (Dong Zhu)

ljding@rcees.ac.cn (Long-Jun Ding)

**
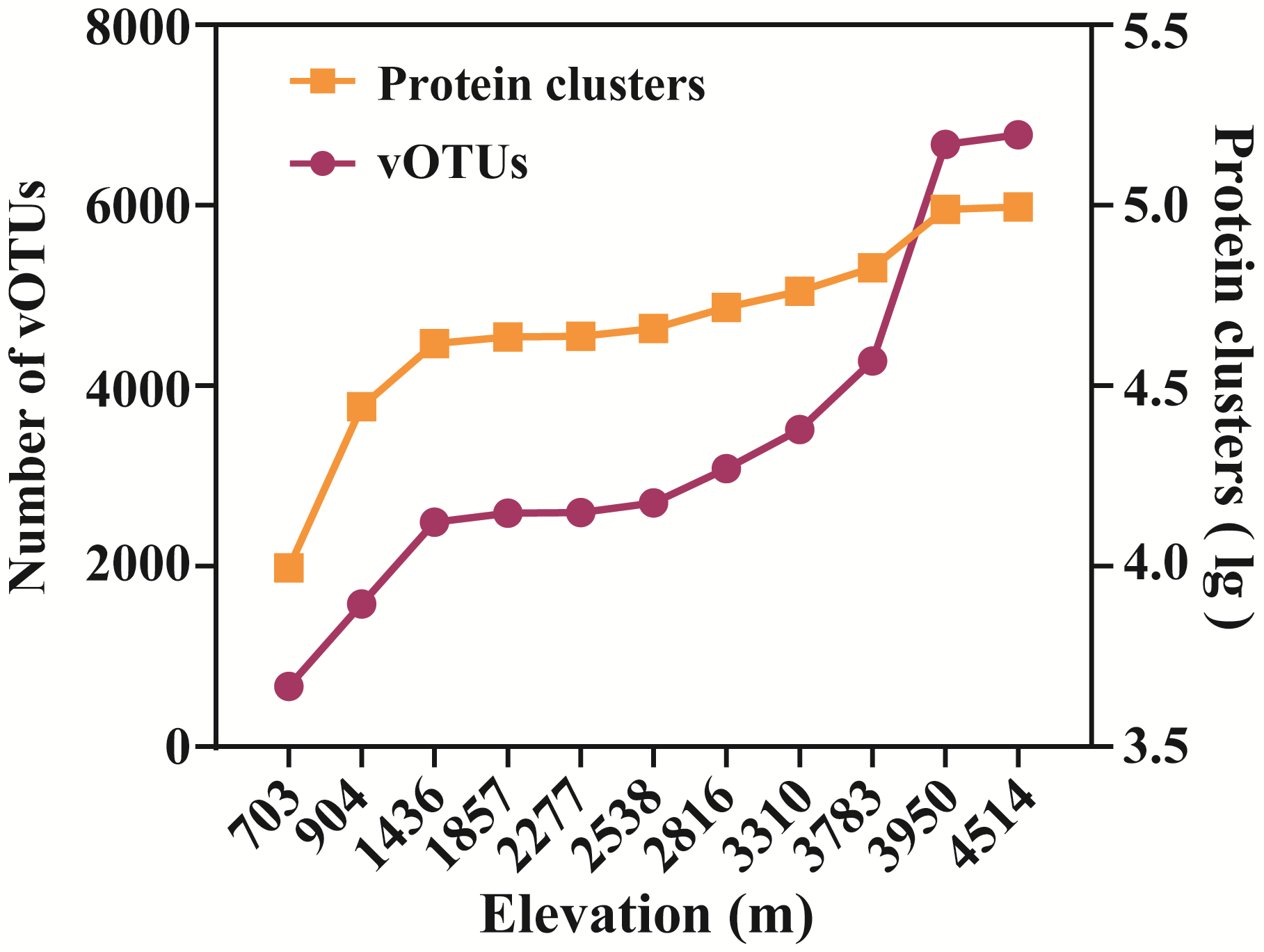
**

**Supplementary Figure 1 Accumulation curves of viral operational taxonomic units (vOTUs, purple) and viral protein clusters (PCs, orange) across elevation gradient.**

**
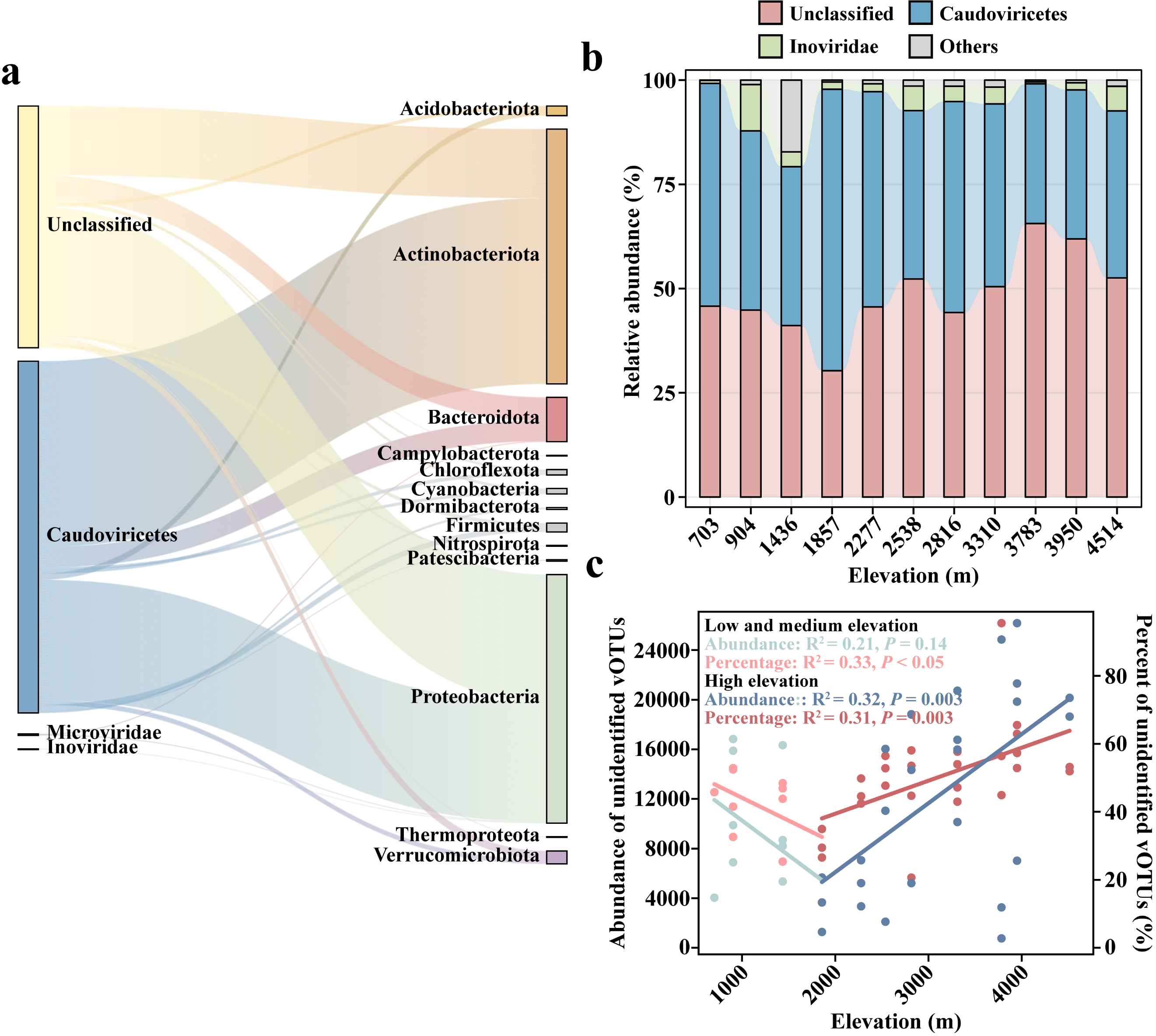
**

**Supplementary Figure 2 Predicted host-virus interactions and the composition of viruses.** (a) The left side displays the percentage and taxonomy of vOTUs with predicted hosts, while the right side presents the taxonomy of those predicted hosts. (b) Relative percentage of vOTUs at the family level at different elevations. (c) Linear regression illustrates the relationship between the abundance and percentage of unidentified vOTUs and elevation.

**
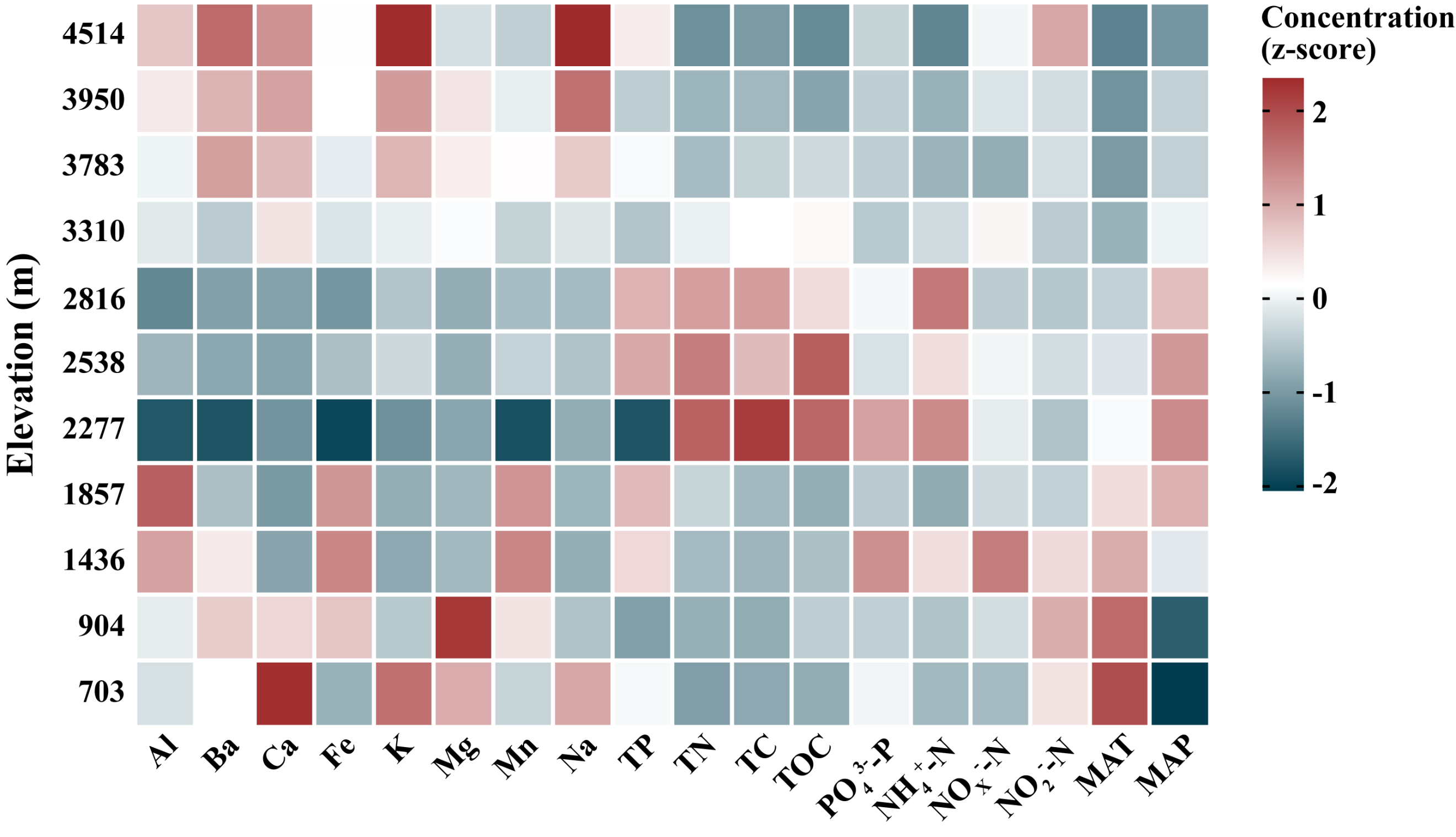
**

**Supplementary Figure 3** **Soil property at different elevations.** The heatmap shows the z-transformed values of the edaphic variables included in the analysis.
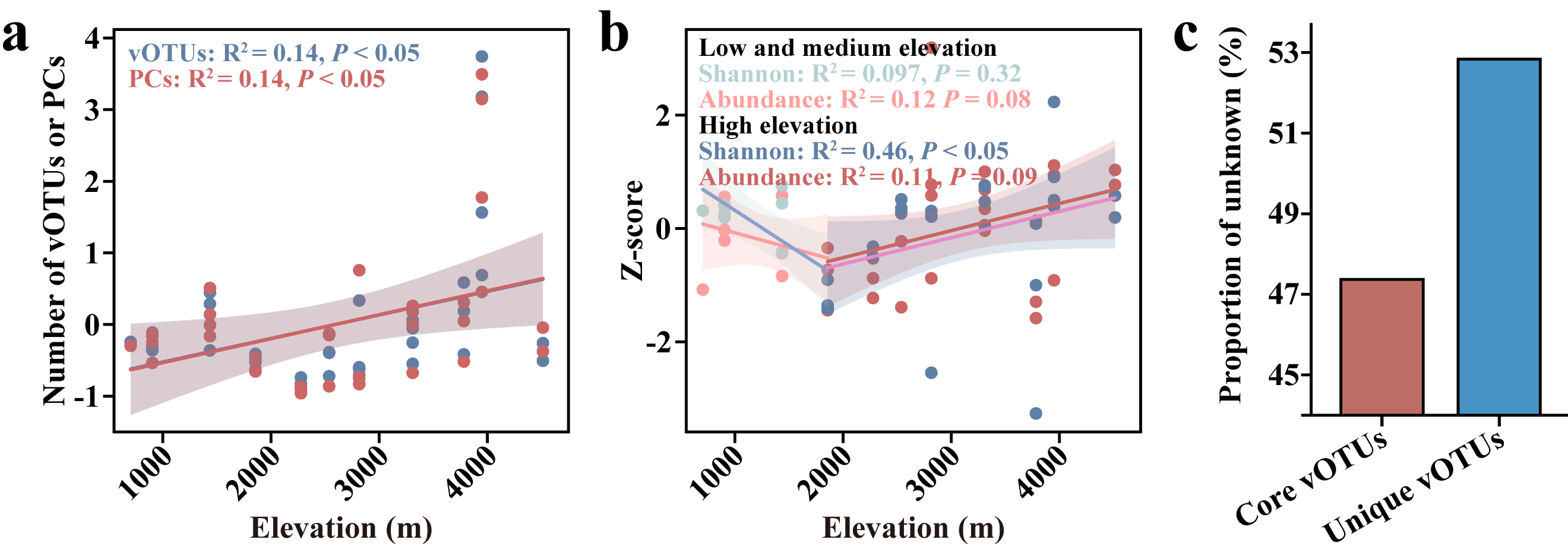


**Supplementary Figure 4 Changes in viral traits across elevation gradients.** Linear regression shows the relationship between (a) the number of vOTUs and PCs, (b) Shannon diversity and the abundance of the viral community, and elevation. (c) Compare unknown ratio between core and unique vOTUs.

**
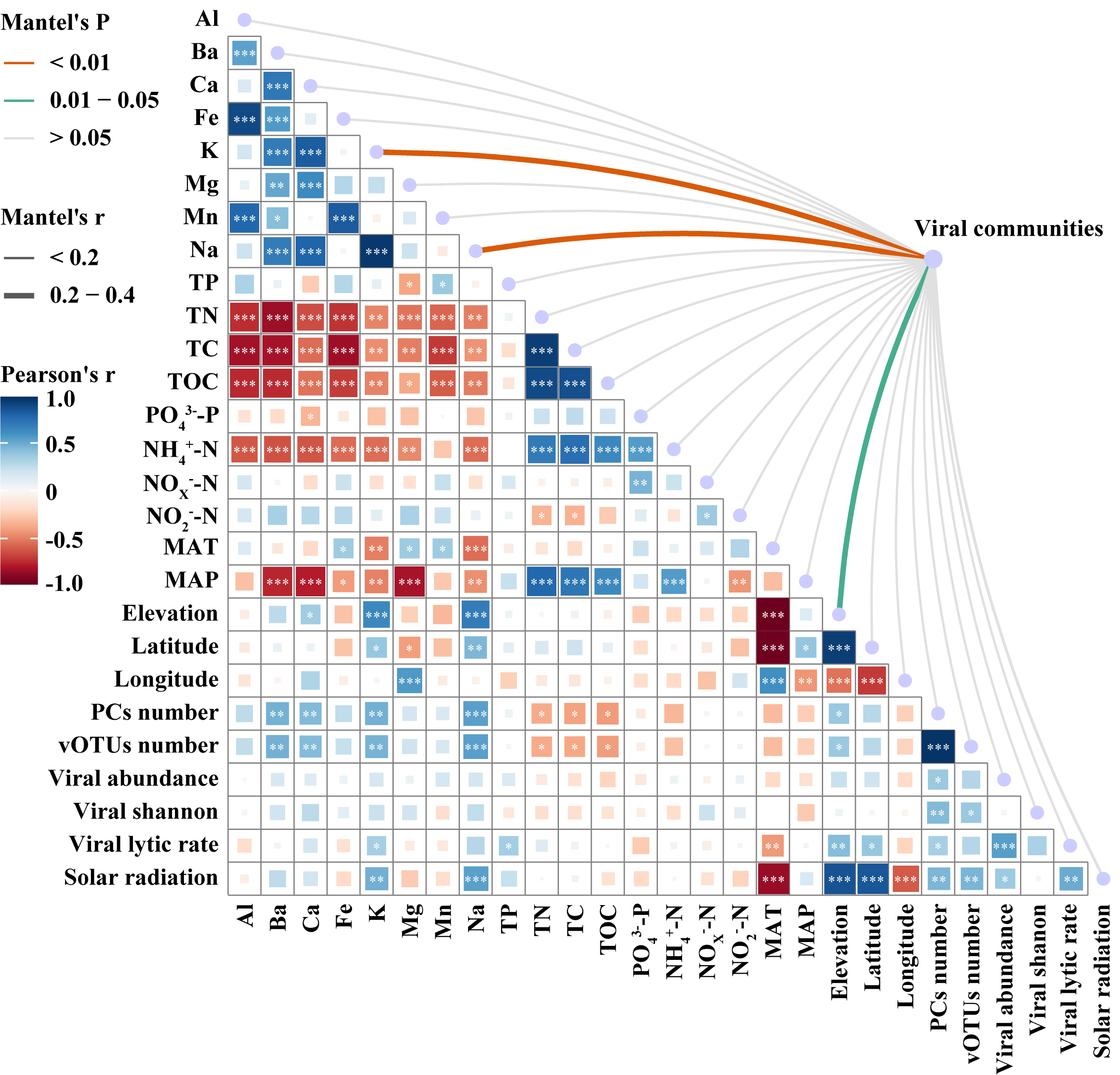
**

**Supplementary Figure 5 Pairwise comparisons of the biotic and abiotic variables.** The color gradient in the heatmap represents Pearson’s correlation coefficients, with an asterisk indicating statistical significance from a two-tailed test adjusted using the Benjamini and Hochberg false discovery rate procedure. Edge width corresponds to Mantel’s r statistic for the respective distance correlations, and edge color reflects their statistical significance.

**
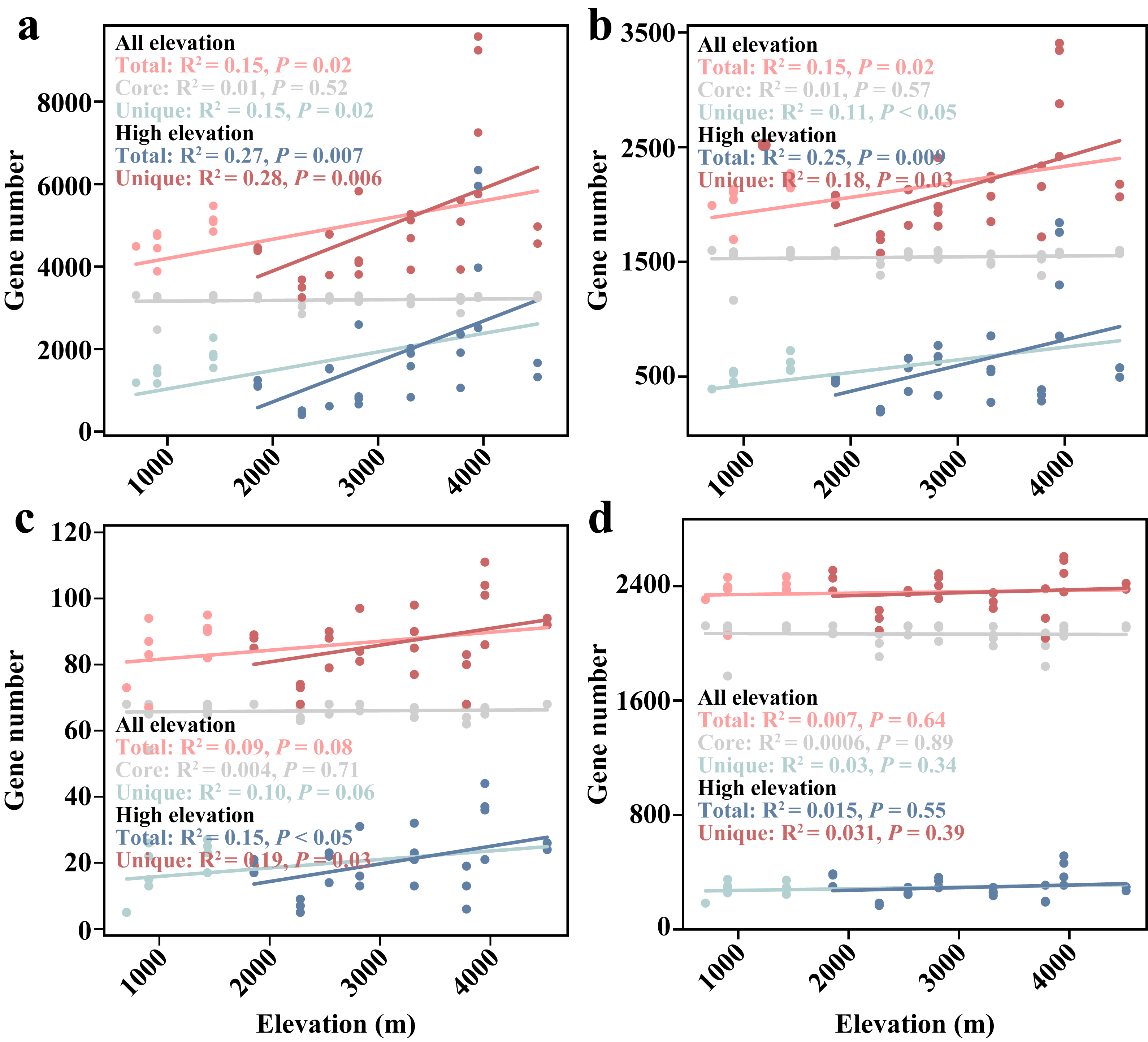
**

**Supplementary Figure 6 The relationship between successfully annotated functional genes in viral proteins and elevation.** Linear regression shows the relationship among the number of successfully annotated functional genes by (a) eggNOG, (b) KEGG, (c) CAZy, and (d) BacMet databases in relation to elevation.

**
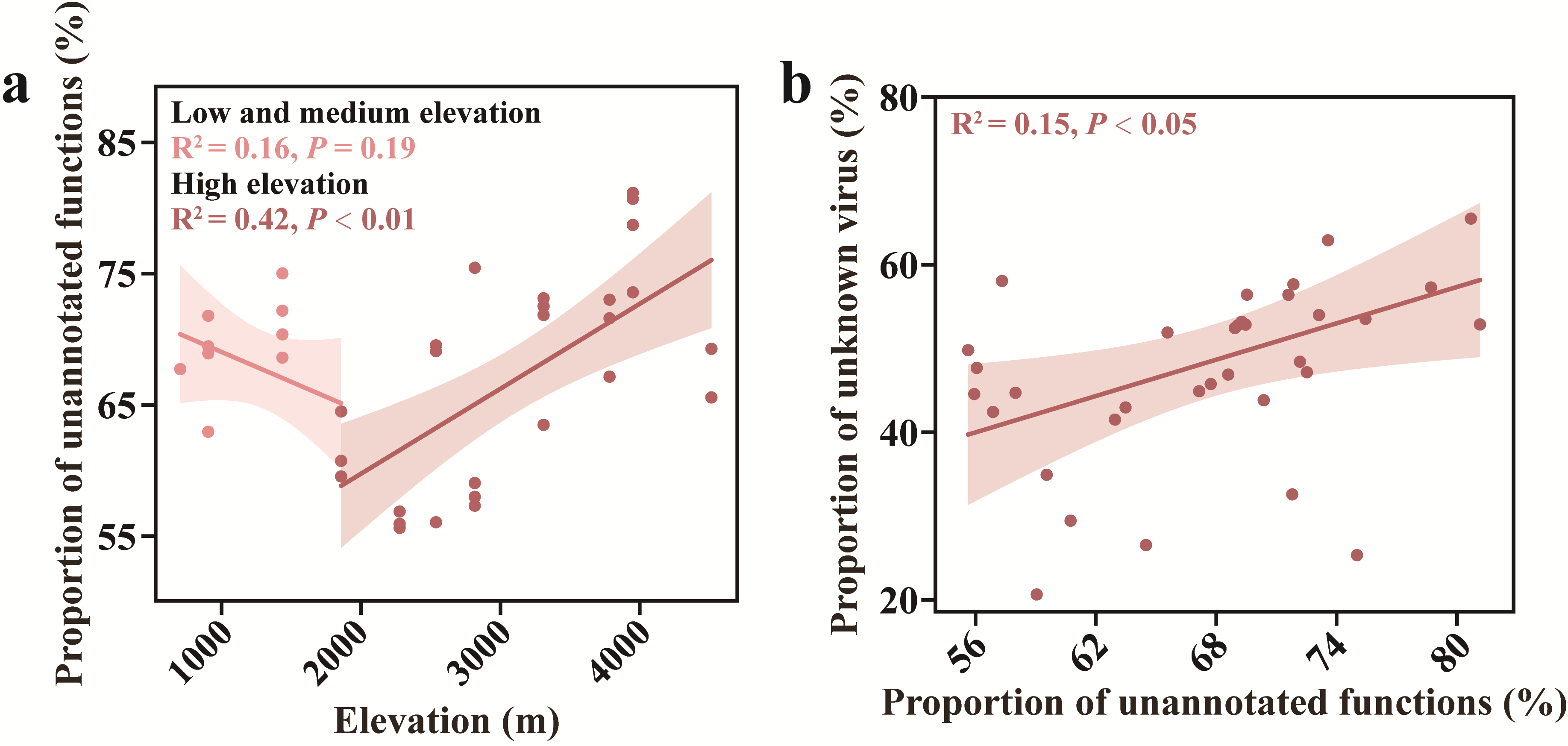
**

**Supplementary Figure 7 The relationship between unannotated functional genes in viral proteins and elevation.** Linear regression illustrates (a) the relationship between the proportion of unannotated functions and elevation, and (b) the proportion of unannotated functions and proportion of unknown virus, respectively.

**
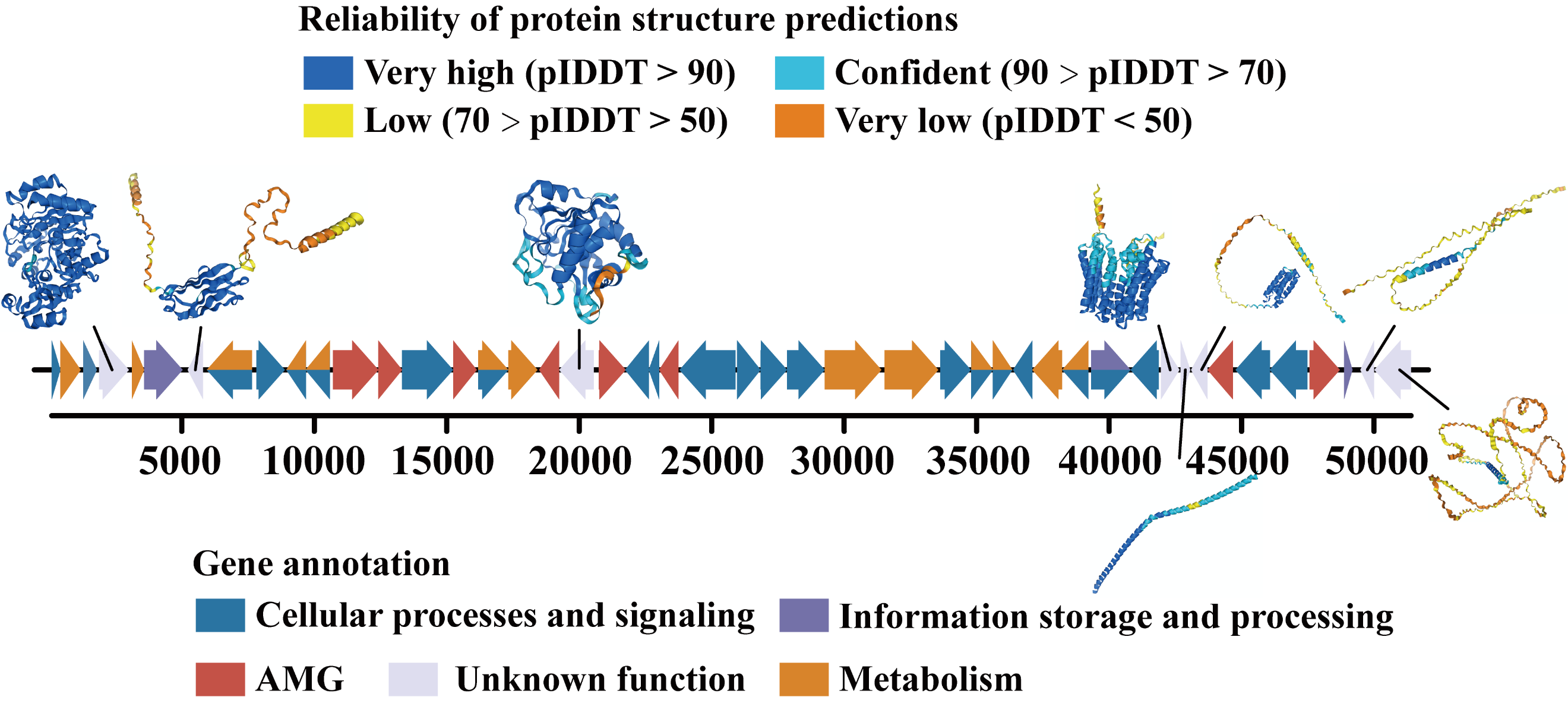
Supplementary Figure 8** **Viral genomic context and unannotated viral protein** **structure.**

**
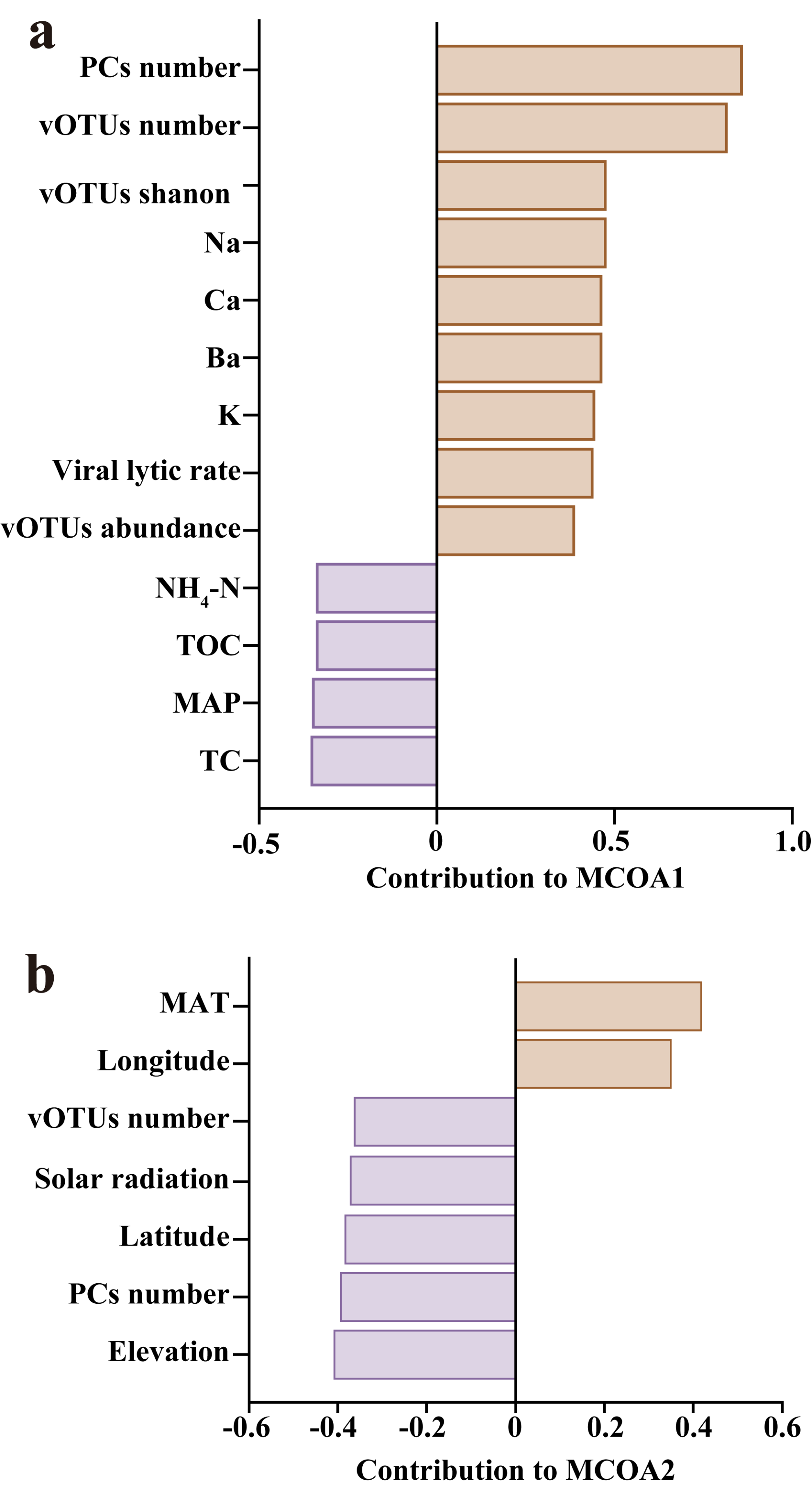
**

**Supplementary Figure 9** **Environmental drivers and viral traits contributions to the MCOA dimensions.** Variable contributions to MCOA dimensions (a) 1 and (b) 2, respectively. Bar colours indicate the direction of the associations between the variable and the MCOA dimensions. Only the variables with significant correlation (*P* < 0.05) with each dimension are reported in this figure.


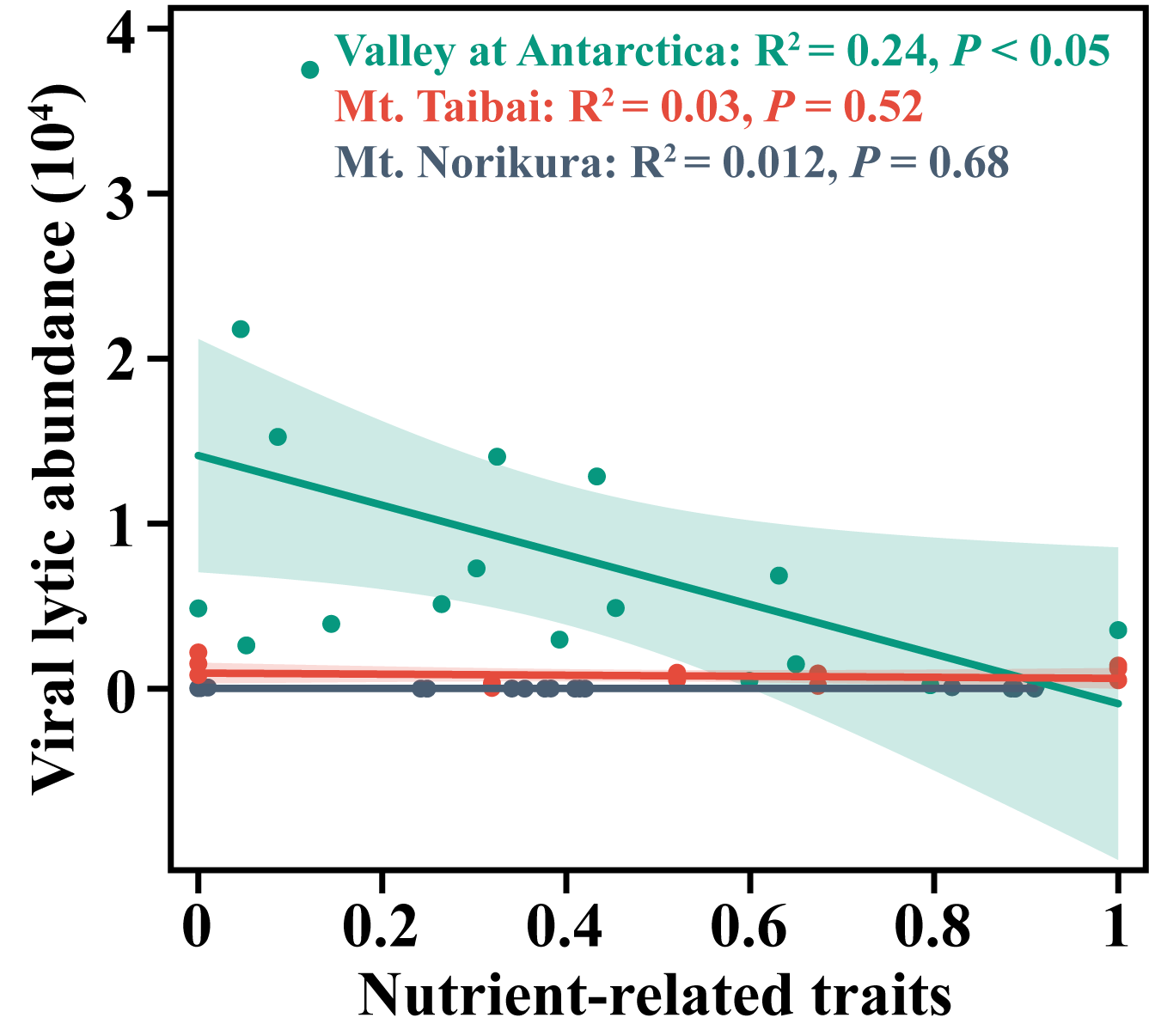


**Supplementary Figure 10 The relationship between viral lytic abundance and nutrient-related traits in Mt. Taibai, Mt. Norikura, and Valley of Antarctica.**


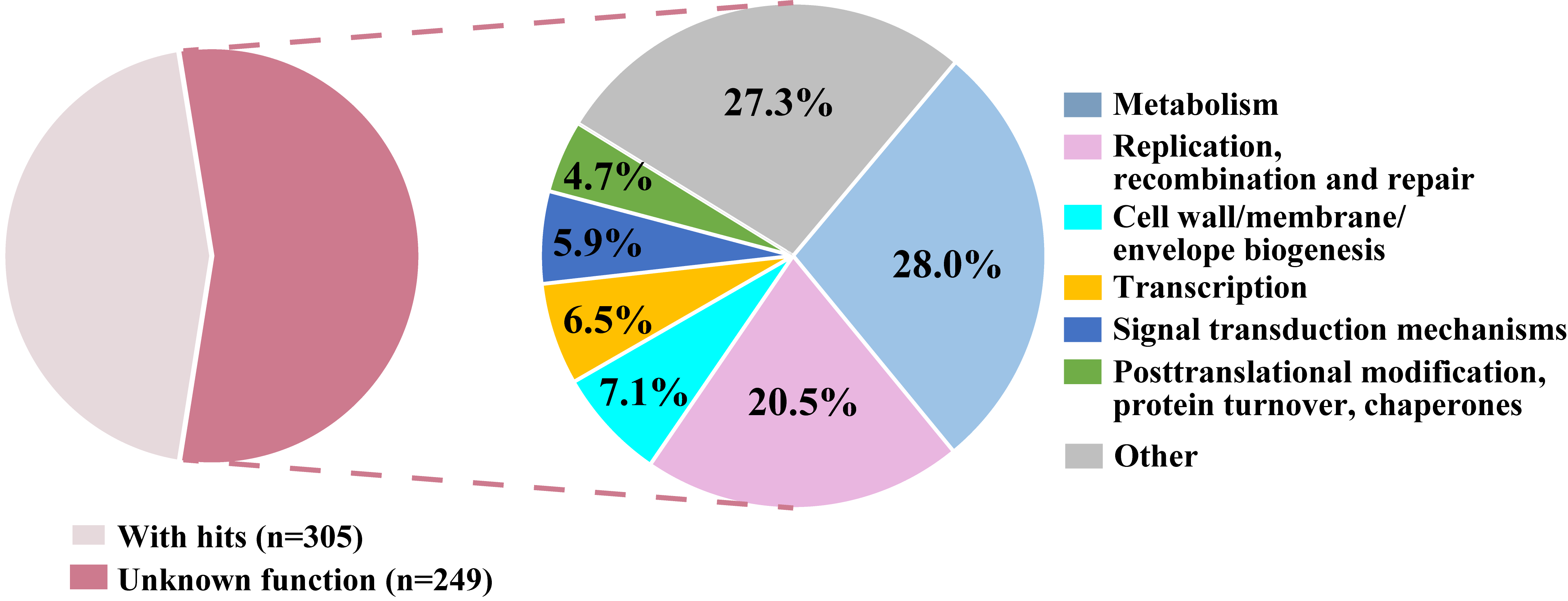


**Supplementary Figure 11 Categories of viral genes under diversifying selection.** Left pie graph, genes under diversifying selection are divided into annotatable and unannotatable groups by comparing to genes in databases. Right pie graph, the annotatable genes from the left pie graph are grouped into different gene categories.


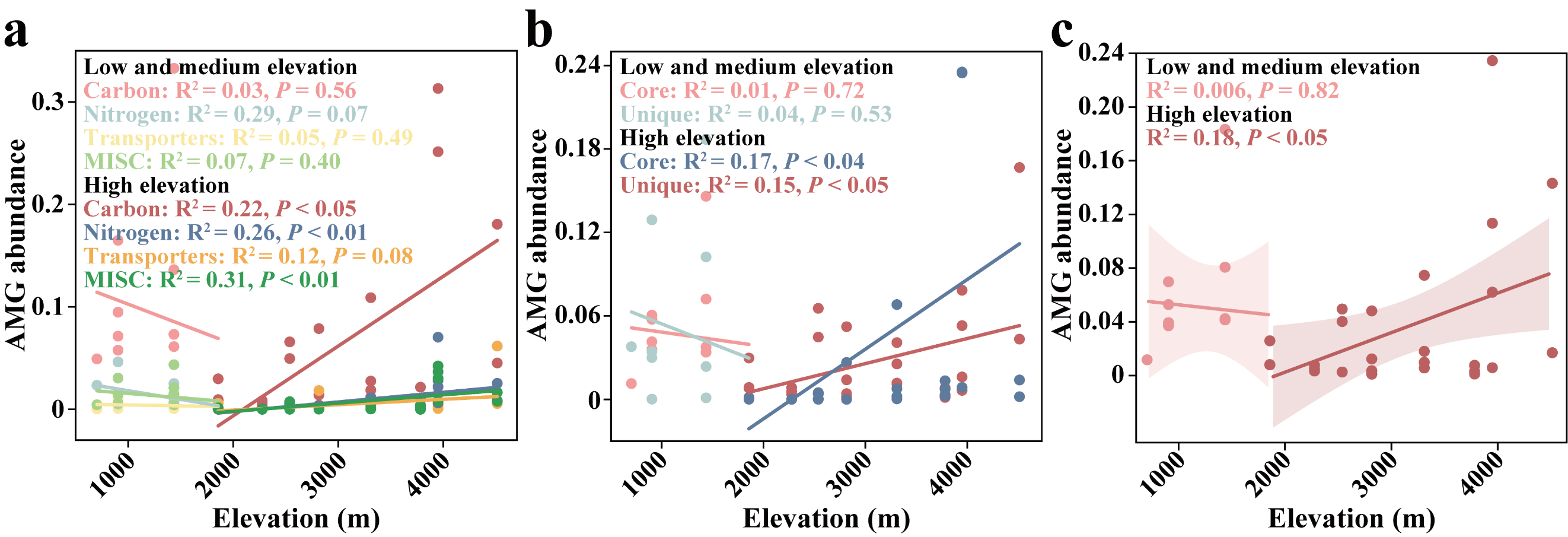


**Supplementary Figure 12 The relationship between viral AMG abundance and elevation in natural soil.** Linear regression reveals the relationships between (a) the abundance of various types of AMGs and elevation, (b) the abundance of core and unique viral AMGs and elevation, and (c) the abundance of key AMGs related to carbon utilization (GT1, GT2, and *glft1*) and elevation.

**
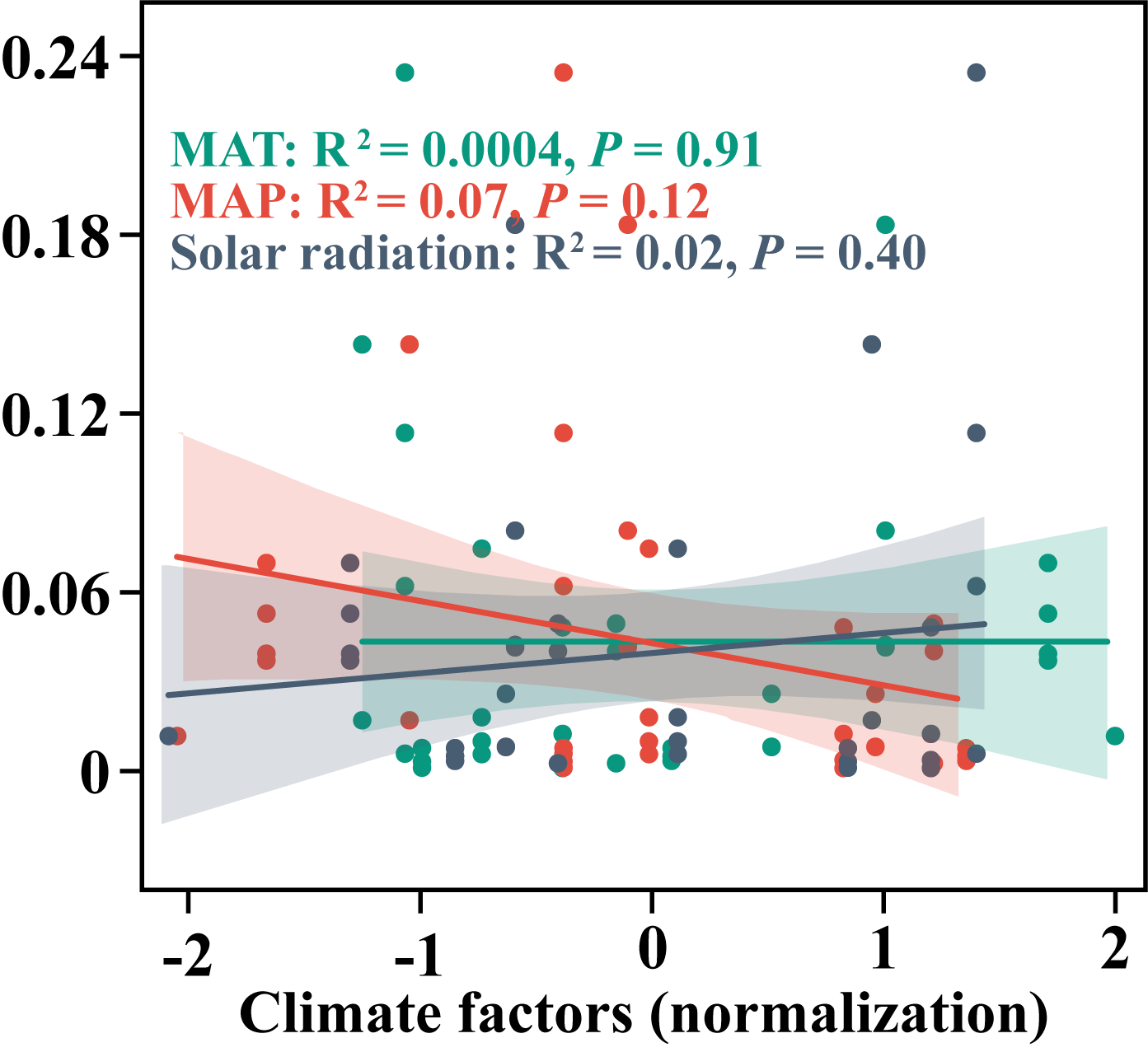
**

**Supplementary Figure 13 The relationship among key AMGs related to carbon utilization (GT1, GT2, and *glft1*), MAP, MAT and solar radiation in natural soil.**

**
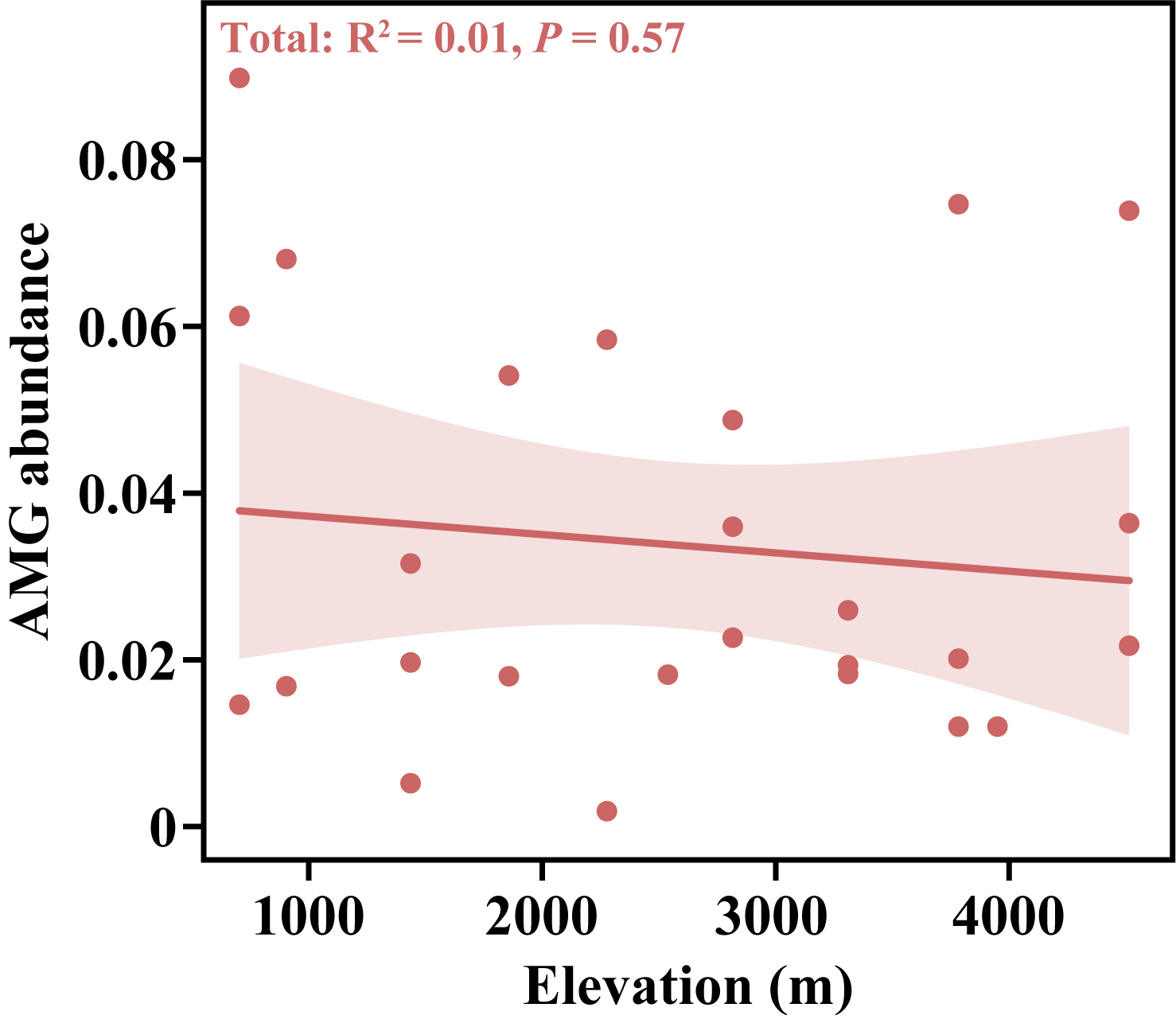
**

**Supplementary Figure 14 The relationship between key AMGs related to carbon utilization (GT1, GT2, and *glft1*) and elevation in** **cultivated soil.**
